# Next Generation Antimicrobial Coatings for High Touch Surfaces: Application, Benefits, Risks, Environmental Impacts and Sustainability Aspects

**DOI:** 10.1002/advs.78521

**Published:** 2026-11-10

**Authors:** Nurshen Mutlu, Peter Askew, Alexander J. Cunliffe, Damjana Drobne, Stefan Fischer, Gillian Iredale, Pascal Kock, Veno Kononenko, Susanne Kurz, Patrick Lichtner, Abby Marchant, Philipp Meier, James Redfern, Giacomo Reina, Simone Schulte, Juliane Spohn, Merve Tunali, Peter Wick, Nihal Engin Vrana, Bernd Nowack

**Affiliations:** ^1^ Empa – Swiss Federal Laboratories for Materials Science and Technology Technology and Society Laboratory St. Gallen Switzerland; ^2^ Industrial Microbiological Services Ltd. (IMSL) Hants UK; ^3^ School of Biological and Chemical Sciences Manchester Metropolitan University Manchester UK; ^4^ Department of Biology, Biotechnical Faculty University of Ljubljana Ljubljana Slovenia; ^5^ Microbial Effects, Coating Additives Evonik Operations GmbH Essen Germany; ^6^ Biological Materials Analysis Group Fraunhofer Institute for Ceramic Technologies and Systems (IKTS) Leipzig Germany; ^7^ Empa – Swiss Federal Laboratories for Materials Science and Technology Laboratory For Nanomaterials in Health St. Gallen Switzerland; ^8^ SPARTHA Medical, CRBS Strasbourg France

**Keywords:** antimicrobial coating, high touch surface, safe and sustainable by design, SSbD, test norm

## Abstract

Antimicrobial coatings for high‐touch surfaces are a promising strategy to reduce the burden of infectious pathogens, limit human exposure, and mitigate transmission in public, healthcare, and other shared environments. This review outlines current antimicrobial coating strategies and their modes of action with attention to their rational applications on high touch surfaces. Because standard laboratory efficacy tests often fail to capture end‐use conditions, the review points to the need for application relevant testing approaches that account for factors such as humidity, temperature, contamination level, inoculum volume, and repeated touching. Beyond efficacy, safe use also depends on how active components are released and on the resulting exposure to humans and the environment, both during their use and at end‐of‐life. Human safety endpoints, such as cytotoxicity, immunotoxicological effects, skin sensitization, and skin irritation, are examined under short‐ and long‐term exposure, in relation to current testing standards and regulatory expectations. In parallel, the review addresses environmental concerns, including potential persistence, bioaccumulation and the transport of released substances into soil, sediments, air and water. Building on these considerations, the review concludes by outlining key principles for applying a safe‐and‐sustainable‐by‐design (SSbD) approach to antimicrobial coatings.

## Graphical abstract

Antimicrobial coatings for high touch surfaces promise infection control but are too often validated only in the lab. This review shows that active‐agent release governs both human toxicity and environmental impact and argues that durability and real‐world performance must be primary design criteria, placing a safe‐and‐sustainable‐by‐design framework (SSbD) at the heart of responsible coating development.

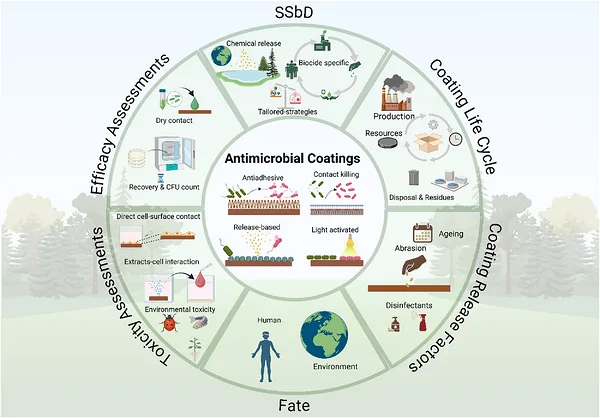

## 1 Introduction

High touch surfaces are recognized as significant vectors for pathogen transmission [1]. These surfaces are commonly found in public transportation systems, schools, and other publicly accessible places. The most critical high touch surfaces can be found in healthcare settings, including waiting rooms, medical equipment and patient‐vicinity areas. These surfaces pose a persistent risk for microbial accumulation and transmission due to constant human contact [1]. Patients have a 20%–40% higher chance of catching a specific pathogen when occupying a room which previously had a patient with the same pathogen [2]. Current mitigation strategies include routine physical cleaning and chemical disinfection using agents like quaternary ammonium compounds, hydrogen peroxide, alcohols and chlorine‐based solutions [3]. However, these methods provide only temporary protection, as surfaces can quickly become recontaminated, allowing microbial populations to accumulate again before the next cleaning cycle [4]. Challenges such as staff shortages, time constraints, inconsistent hygiene practices, and the high cost of sustained disinfection protocols further complicate effective management [4]. For instance, monkeypox virus (MPXV) can potentially be transmitted through dermal contact with contaminated high touch surfaces such as glass, stainless steel and plastic for extended periods of time, reinforcing the need for more robust and long‐lasting hygiene interventions [5, 6]. Additionally, concerns about the environmental impact and practical limitations of overusing conventional cleaning agents necessitate the development of more durable, self‐sufficient hygiene solutions that can provide continuous protection, reduce costs, and minimize environmental impact without frequent human intervention [7, 8, 9, 10].

In this context, antimicrobial coatings have progressively become a promising complementary approach to traditional hygiene practices for high touch surfaces [4]. Advances in material science have enabled the development of high performance, multifunctional materials with bioactive properties that help prevent pathogen contamination and limit the spread of infections [11]. Yet, designing efficient antimicrobial coating systems remains a challenge, as contamination and biofilm formation on high touch surfaces continue to represent a significant medical, economic, and social burden.

The design strategies of antimicrobial coatings can be broadly categorized in three dominant systems, namely antiadhesive coatings, antimicrobial agent releasing systems, and contact‐killing surfaces [12]. Each strategy, however, faces its own limitations. Antiadhesive surfaces are prone to fouling by organic matter and dead cells, reducing their effectiveness over time, and they often exhibit poor resistance to mechanical or chemical stress [13]. Additionally, imperfections in the coating can serve as nucleation sites for microbial attachment. Contact‐killing surfaces require close proximity between microbes and the surface and often demand defect‐free fabrication, making large‐scale production technically challenging [12]. Releasing systems are influenced by the physicochemical properties of their environment, such as medium composition or pH which can alter degradation profiles and release kinetics. These coatings can also rely on moisture to activate efficiently [14] and pose toxicity and environmental concerns due to uncontrolled leaching of active compounds [12, 13, 15].

To address these limitations, novel strategies are increasingly focused on hybrid systems that combine multiple mechanisms (e.g., release‐based + contact killing, or release‐based + nanostructured systems) and stimuli‐responsive coatings [8, 12]. Of particular interest are light activated antimicrobial coatings, which offer on‐demand activation and may reduce the risk of resistance development [3]. However, identifying promising candidates remains challenging because it depends on how antimicrobial efficacy is assessed, as the performance is highly influenced by environmental conditions, contamination modes, and usage patterns.

Moreover, as these novel materials become more complex, standardized tests may no longer be sufficient to predict real‐world efficacy. For antimicrobial coatings to become a realistic complement to existing hygiene practices, their performance should extend beyond laboratory‐based antimicrobial efficacy. High touch surfaces in public transport, and healthcare environments, such as door handles, handrails, waiting areas and near patient surfaces are exposed to frequent contact, routine cleaning and aesthetic and regulatory requirements. Therefore, developed antimicrobial technologies should ideally be compatible with established coating formulations including two component polyurethane or acrylics or UV curable systems and applicable through conventional methods such as spray coating, dip coating, brushing or roller applications [16, 17]. Approaches that require major changes in formulation, substrate preparation, curing conditions, or application infrastructure may face substantial barriers to adoption, regardless of their antimicrobial activity. This highlights the need for the evaluation of antimicrobial coatings not only in terms of efficacy but also with respect to durability, cleanability, process compatibility, and practical scalability. Many of these coatings therefore require adapted or complementary evaluation methods that can assess the performance under realistic conditions, since their modes of action differ substantially and performance requirements vary depending on the intended use case.

Equally important to antimicrobial efficacy are human health and environmental safety aspects [18, 19]. As the use of antimicrobial coatings expand, particularly those involving active components like metal ions, biocides, or nanomaterials, concerns about unintended release and long‐term impacts are increasing [4, 7, 18, 20]. For instance, silver ions leaching from antimicrobial textiles during washing [21] or the release of nano‐ and microparticles from coated surfaces due to mechanical abrasion have been reported [7]. Such evidence suggests that antimicrobial components can enter environmental compartments during product use, potentially posing ecotoxicological risks [7, 22]. In addition, human exposure can occur via dermal contact, inhalation of particles or aerosols generated during use, and inadvertent ingestion through hand to mouth transfer from the surfaces. Considering these exposure pathways is essential for understanding the short‐ and long‐term safety of the coatings. To mitigate these concerns, it is critical to evaluate the likelihood and consequences of material release, environmental persistence, the (eco)toxicity of degradation products, and occupational exposure, ideally from the earliest stages of coating design [18, 19].

Traditional chemical safety assessments are often resource‐intensive and not easily adaptable to the complexity of novel materials. These assessments are typically applied at a late stage of development, which can lead to the need for redesign if safety concerns arise, resulting in delays and increased costs. Environmental sustainability is another important aspect that should be considered when designing new materials and coatings, highlighting the growing need for a more proactive, and integrated approach toward safety and sustainability [23]. The Safe and Sustainable by Design (SSbD) framework by the European Commission offers a solution by promoting the incorporation of safety, and environmental compatibility from the outset of material development [24, 25, 26]. Importantly, SSbD encourages a holistic evaluation which supports not only the development of “greener” materials/chemicals by design principles, but also assessment of their long‐term safety and sustainability. The application of this framework can lead to the development of effective yet safe and sustainable antimicrobial surfaces.

This review provides a cross‐disciplinary perspective on novel antimicrobial coatings on high touch surfaces. First, we summarize major antimicrobial coating strategies and their mechanism of action, with emphasis on innovations beyond traditional approaches. Second, we examine current antimicrobial testing methods and discuss their relevance and limitations for predicting performance under realistic use conditions. Third, we discuss the potential release of active ingredients, particles and degradation products together with the associated human health and environmental exposure pathways. Finally, we integrate these considerations within the SSbD framework to highlight how antimicrobial coating development can be guided toward technologies that are effective, durable, safe and environmentally responsible.

## 2 Current Concepts and Strategies for Antimicrobial Coatings

Conventional disinfection strategies rely heavily on repeated human intervention and are often limited by inconsistent application and short‐term effectiveness. To address these shortcomings, surface engineered antimicrobial coatings have been developed to provide continuous protection against microbial contamination without active user involvement. Based on their mode of action, antimicrobial surface strategies can be broadly categorized into passive, active, and hybrid approaches. Passive surfaces aim to repel pathogens and prevent their adhesion by tailoring surface chemistry or topography. Active surfaces, in contrast, are designed to kill or inactivate microorganisms through mechanisms including membrane damage, producing reactive oxygen species and disturbing key cellular pathways. Hybrid systems on the other hand integrate multiple passive and/or active strategies to achieve synergistic antimicrobial effects, which use particular advantages of various strategies for high touch surfaces exposed to repeated contamination. In the following sections, these strategies are discussed in terms of their underlying mechanisms, advantages, limitations, along with innovative examples in the field.

### 2.1 Passive Strategies

Passive antimicrobial concepts work by modifying surface properties to create conditions that prevent adhesion and colonization without actively killing the microorganisms [27, 28]. These concepts rely on antiadhesive surface modifications, topographical features, and non‐fouling coatings that make surfaces inhospitable to microbial attachment. Microbial load on the surface increases through the attachment and subsequent proliferation of microorganisms, with surface attachment representing the critical first step in this process. This attachment mainly occurs through the interaction between microorganisms with the adsorbed layers of organic contamination of the surface. The rationale behind antiadhesive coatings is that, by preventing this initial attachment of microorganisms, significant microbial accumulation can be avoided, thereby reducing the potential for transmission.

This strategy is widespread in nature and has evolved independently in plants and animals as an effective means of maintaining surface cleanliness. Well‐known examples include butterfly wings and lotus leaves, although similar surface architectures are found in other insect species, fish, birds, and flowering plants. This effect is mainly achieved by intricate micro and nanoscale structures that can result in superhydrophilic or superhydrophobic behavior, which limits the adhesion of organic and inorganic contaminants and subsequently that of bacteria [29].

Beyond disruption of the cell wall, recent work also sheds light on the underlying mechanisms of surface structures. The advances in micro and nanoengineering technologies have enabled the development of biomimetic micro/nano‐structured surfaces (Figure 1), a growing strategy for antifouling applications. For example, nanopillars mimicking dragonfly wings have been shown not only to interfere with bacterial adhesion but also to impede bacterial division and induce significant oxidative stress [30]. Despite these promising effects, many micro/nanostructured surfaces require specialized fabrication equipment and are often limited in terms of scalable production and surface geometry. Nevertheless, recent approaches, such as roughness control through the deposition of nanofilaments, offer elegant ways to overcome these limitations [30, 31].

**FIGURE 1:**
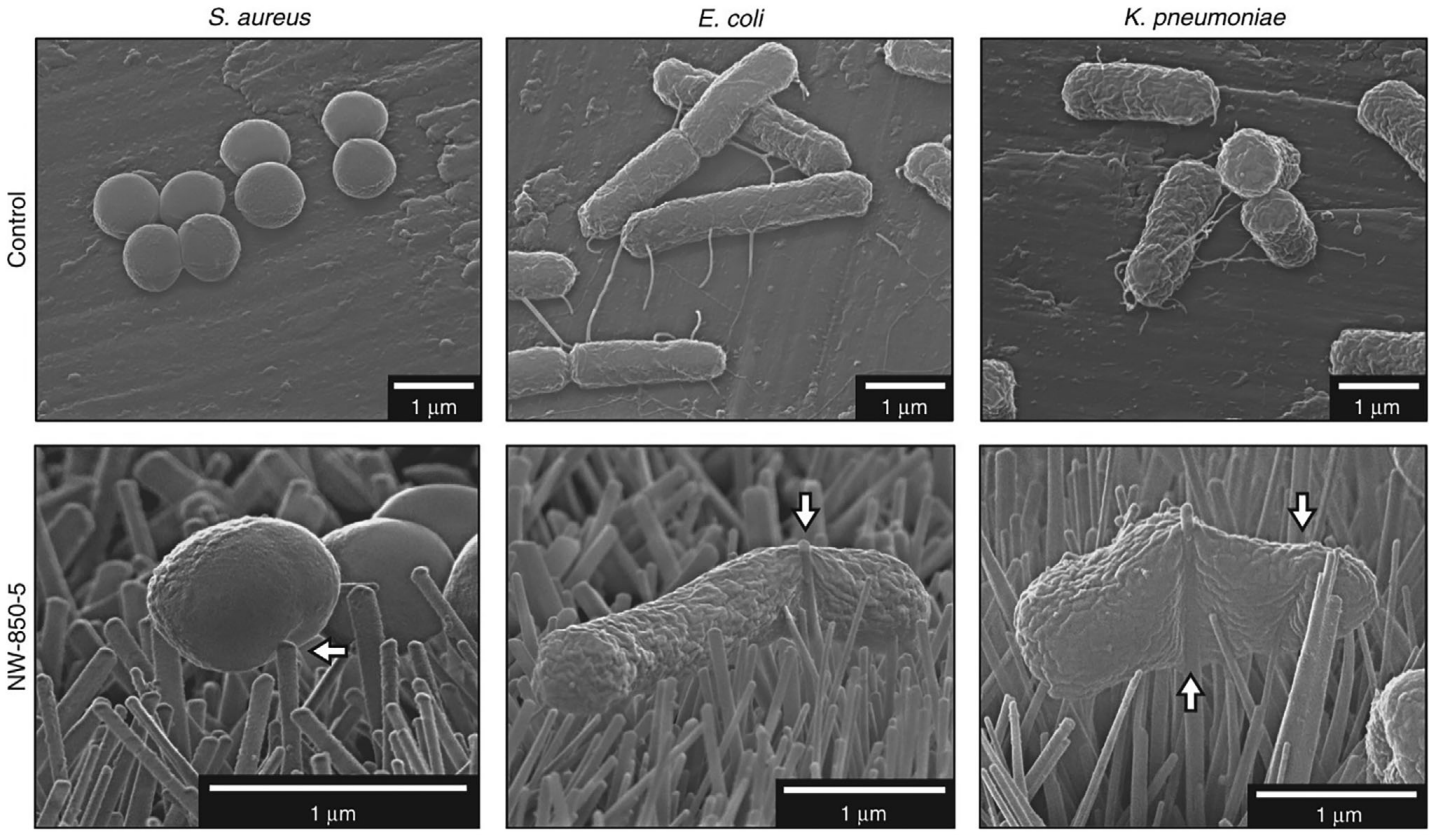
Effect of nanostructured surfaces on bacterial membranes on three pathogenic bacteria (*Escherichia coli*, *Staphylococcus aureus* and *Klebsiella pneumoniae*). SEM images compare bacterial attachment on flat titanium alloy surface (control) and nanopillar surface (NW‐850‐5). White arrows indicate nanopillar induce envelop deformation regions. (Reproduced from Jenkins et al. [30], Copyright 2020, Springer Nature, licensed under Creative Commons Attribution by 4.0).

From a scalability and practical application perspective, polymer‐based coatings represent a more accessible route to antifouling surfaces. Superhydrophilic polymers such as polyethylene glycol (PEG) and poly vinyl alcohol (PVA) are commonly used due to their relative bioinertness and ability to form gels. In contrast, the use of superhydrophobic surfaces is more limited due to health and sustainability concerns related to the commonly used hydrophobic agents such as per‐ and polyfluoroalkyl substances (PFAS), but there is also active research going on for the development of new polymers and composites for similar effects [32].

Surfaces have also been developed that can simultaneously harness the antifouling effects of surface topography, roughness, and chemical structure [33]. One such system which uses surface porosity and wettability in combination is SLIPS (slippery liquid infused porous surfaces). SLIPS surfaces harness the surface microporosity with specific lubricants which render the efficacy of the surface against fouling even to complex fouling agents such as marine species larvae [34]. Although mainly limited to marine applications, green SLIPS systems are under development [35] that can be applied in more constrained applications such as hospital environments. Another example is hierarchical structures consisting of polydimethylsiloxane particles at the micrometer scale, structural wrinkles at the tens of micrometer scale and gold nanoparticles at the nanoscale to obtain omniphobic coatings applicable to various surfaces [36]. Similarly, Ladouceur et al. [37] developed a hierarchical coating from wrinkled polysiloxane nanostructures using n‐propyltrichlorosilane on activated polyolefin, demonstrating a 1.2 log reduction in culturable E. coli transferred to the surface, using ∼5.7 × 10^7^ CFU mL^−1^ inoculum concentration [37]. Despite their effectiveness in reducing microbial attachment, antiadhesive coatings do not possess intrinsic bactericidal activity; organisms that fail to adhere remain viable and may be redistributed rather than eliminated. In high‐touch surfaces, this is partly mitigated since non‐adherent organisms remain more susceptible to removal than biofilm [38], but antiadhesive coatings are regarded as a complementary strategy rather than a substitute for an active strategy.

### 2.2 Active Strategies

#### 2.2.1 Contact Killing Coatings

In applications where antifouling effects alone are not sufficient; a second potential route of antimicrobial effect is through contact killing. Contact killing refers to the physical disruption or inactivation of microorganisms upon direct contact with the surface without intentional release of any antimicrobial agents. This strategy is particularly suitable for applications where the limitation of the transfer of the bactericidal agent is critical, such as touch surfaces [39] or food contact surfaces in food processing and packaging environments, where regulatory restriction on the releasing of substances into food favor non‐releasing, contact active strategies [40]. This will limit unintentional transfer of active ingredients which can be dangerous for the people that come into contact with the coated surfaces. Moreover, contact killing mechanisms in contrast to release based ones minimize the release of active agents therefore reduce the likelihood of exposing bacteria to subminimum inhibitory concentration which is a strong driver of resistance development [41]. Contact killing systems are also more advantageous over release‐based applications when there is limited contact with liquids including dry surfaces and airborne bacteria [42] (Figure 2).

**FIGURE 2:**
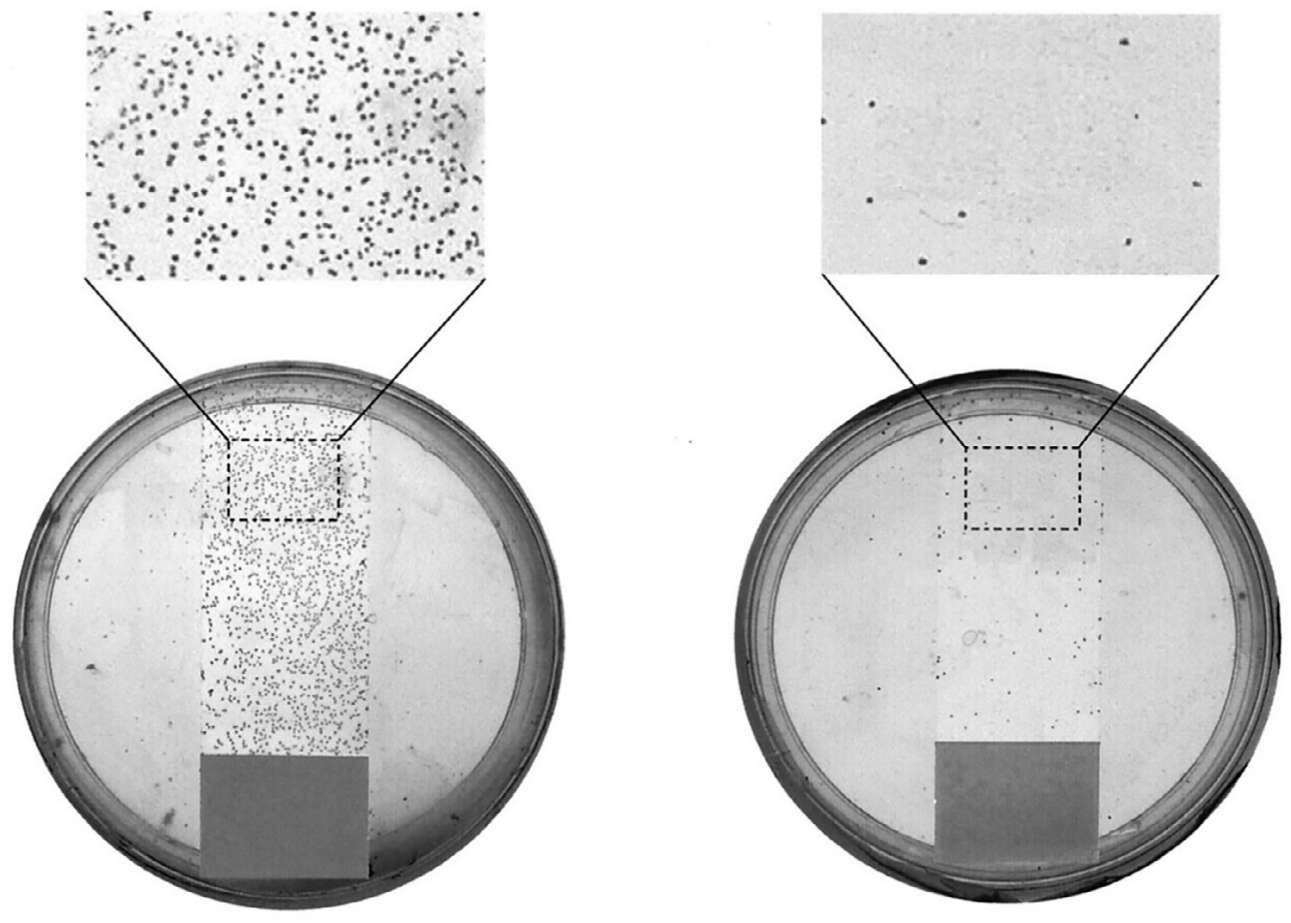
Contact killing activity of Poly(4‐vinyl‐N‐alkylpyridinium bromide) on glass surface. The unmodified glass enables the adhesion and proliferation of bacteria, whereas the modified surface kills bacteria via contact killing. (Reproduced from [42], Copyright 2001, National Academy of Sciences).

Contact killing can be achieved by surface immobilized metals such as silver and copper [43], quaternary ammonium [44] and other antimicrobial molecules immobilized on the surface [45], or surfaces that can induce Reactive Oxygen Species (ROS) generation [46]. For instance, Ghosh et al. [15]. reported non‐releasing, sprayable coatings based on quaternary small molecules that could be applied to a wide range of substrates, including nylon, polyethylene, and surgical masks. The coating exhibited a strong inactivation of high microbial loads, including MRSA, fungi, H1N1 influenza virus, and SARS‐CoV‐2, primarily through membrane disruption and disintegration within 30 min of exposure. Importantly, the coating retained antimicrobial efficacy after washing with ethanol, indicating covalent attachment of the active molecules to the substrate [15].

Additional to immobilized systems, contact killing strategies can also be achieved through engineered surface topographies. While these approaches share some conceptual similarities with antifouling surfaces, their designs are specifically optimized to disrupt, stretch, or pierce bacterial membranes rather than to prevent adhesion. An offshoot of this approach are mechanobactericidal surfaces where the topographical properties are reinforced with a deformation behavior to improve contact‐based bactericidal activity [47]. For instance, Seth et al. [48]. developed ZnO nanostructured surfaces which are spin‐coated with an ionic liquid (DMIM‐BF_4_) on silicon wafers. The nanostructured surfaces showed a significant decrease in *E. coli* viability, especially for nanofibrous structures. Coating with ionic liquids showed further reduction in the bacterial colony formation [48]. Ionic liquids have been identified as emerging antimicrobial agents for advanced bactericidal industrial surfaces [49].

Despite these advantages for certain applications, contact killing systems exhibit inherent limitations. A key challenge is fouling of the surface: as most of the contact killing antimicrobial coatings do not have an antifouling effect, they can be easily covered by organic and inorganic contamination, potentially rendering the coating inactive after a given period of time. This aspect is especially relevant in environments where surfaces are exposed to biological fluids or repeated contamination.

#### 2.2.2 Release‐Based Systems

Release‐based systems rely on the controlled delivery of active components from a surface into the surrounding environment to inhibit or kill microorganisms over time. Unlike passive or contact killing strategies, their antimicrobial efficacy depends on the sustained availability of the released agent at concentrations sufficient to suppress microbial growth. These systems are particularly attractive for applications requiring prolonged antimicrobial activity or repeated protection against multiple contamination events.

Historically, release‐based systems have been widely applied in a biomedical context. One of the most established examples are the antimicrobial pouches for pacemakers which need to be active for months [50]. The available solutions are systems that can actively release antimicrobial agents for a prolonged period of time for efficacy against multiple pathogen contaminations. Such systems are suitable for cases like hernia meshes, especially those in contact with the gastrointestinal system and its microbiota, because they can be exposed to high bacterial loads repeatedly and are required to address multiple contaminations [51]. While these examples originate from implantable devices, they illustrate general design principles of releasing systems that are also relevant to other long‐term antimicrobial applications.

In the context of high touch surfaces, several release‐based systems have been developed aiming to combine sustained antimicrobial efficiency with practical applications. For instance, Saud et al. [52]. reported a smart release system based on Cu^2+^‐loaded cation exchange resin which is dispersed in poly(vinyl‐co‐butyral‐co‐vinyl alcohol‐co‐vinyl) acetate binder and applied to stainless steel. Cu^2+^ release occurred via ion exchange mechanisms, resulting in significant inactivation of SARS‐CoV‐2 within four hours. Compared to metallic copper dispersed in organic coatings, this system demonstrated improved antiviral performance and was proposed as a cost‐effective candidate for high touch surfaces [52]. Similarly, sprayable coatings based on the release of Ag^+^ ions from immobilized mesoporous silica nanoparticles exhibited strong antibacterial activity against multiple pathogens, including *Staphylococcus aureus, Escherichia coli* and *Candida albicans* [53]. In another example, Ag‐doped phosphate glasses were incorporated as antimicrobial additives into a powder coating applied to aluminum substrates [54]. These coatings are already in commercial use on public surfaces in hospitals, schools, meeting rooms, and door handles. To assess real world performance, the study evaluated antimicrobial efficacy under simulated indoor weathering and repeated human contact using artificial sweat and humidity cycles [54]. Although silver was not detected on the immediate surface, it was released at very low, safe concentrations sufficient to significantly reduce bacterial viability. The results indicated that antimicrobial efficacy depended more strongly on bacterial strain and additive concentration than on the degree of surface weathering, and that oxidizing cleaning agents could further enhance hygienic performance by increasing surface wettability [54].

Release‐based systems can be designed using several mechanisms. Passive diffusion of antimicrobial agents from polymeric matrixes is commonly employed particularly for antibiotics or metal ions [51]. For hydrophilic antimicrobial molecules such as some antimicrobial peptides, sequestration within hydrogels is another potential route of release. Additional strategies include polymer matrix degradation via hydrolytic or enzymatic pathways, leading to controlled release, as well as stimuli‐responsive systems that respond to environmental cues such as pH changes [55] or the presence of specific bacterial molecules [56]. Together, these approaches allow tailoring of release profiles to specific applications but also introduce additional design complexity. One of the central challenges of releasing systems is control over the release kinetics. In many cases, an initial burst release results in a significant decrease in the antimicrobial agent reservoir and diminishes long‐term antimicrobial activity. An optimal control of particle size for (nano)particle‐based release‐systems was found to be key for prolonged activity with the lowest possible loading [57].

Another critical limitation is potential downstream environmental issues linked to the effect of released compounds on ecosystems. The release systems are also more prone to generating concentration gradients where subminimum inhibitory concentration (sub‐MIC) zones can facilitate resistant bacteria development. For high touch surfaces, these limitations are especially critical, as frequent contact and cleaning can accelerate agent depletion while increasing the risk of human and environmental exposure. From a regulatory perspective, release‐based antimicrobial coatings present specific challenges that are particularly relevant for high touch surface applications. While migration limits and standardized testing approaches are well established in the EU for materials intended to come into contact with food [58], no equivalent regulatory thresholds currently exist for antimicrobial coatings used on indoor hygienic surfaces [54]. In addition, standardized methods to quantify the release of active components from coatings under realistic use conditions are lacking, which complicates comparison between technologies and the assessment of released amounts against existing regulatory benchmarks [54]. These considerations highlight that, despite their maturity, release‐based systems must be carefully adapted to high touch surface applications to balance efficacy, durability, and safety.

Recent developments place the focus increasingly on combining release‐based systems with additional protective mechanisms. Hybrid designs incorporating contact killing functionality, antifouling structures, or multilayer architectures have been reported to mitigate some of the inherent limitations of purely releasing systems [59]. For example, Zou et al. reported a sequential structure, three lines of defense coating (Figure 3), against *Pseudomonas aeruginosa* which combines quaternized chitosan based bactericidal activity, acylase based quorum sensing inhibition and hyaluronic acid antiadhesive top layer. By targeting bacterial adhesion, survival and biofilm maturation simultaneously, the coating reported to reduce attached bacteria by 98% (∼1.7 log) using 500 µL, 1 × 10^7^ CFU mL^−1^ with 4 h and suppressed biofilm development over three days highlighting the potential of multifunctional architectures over purely release based antimicrobial strategy [59].

**FIGURE 3:**
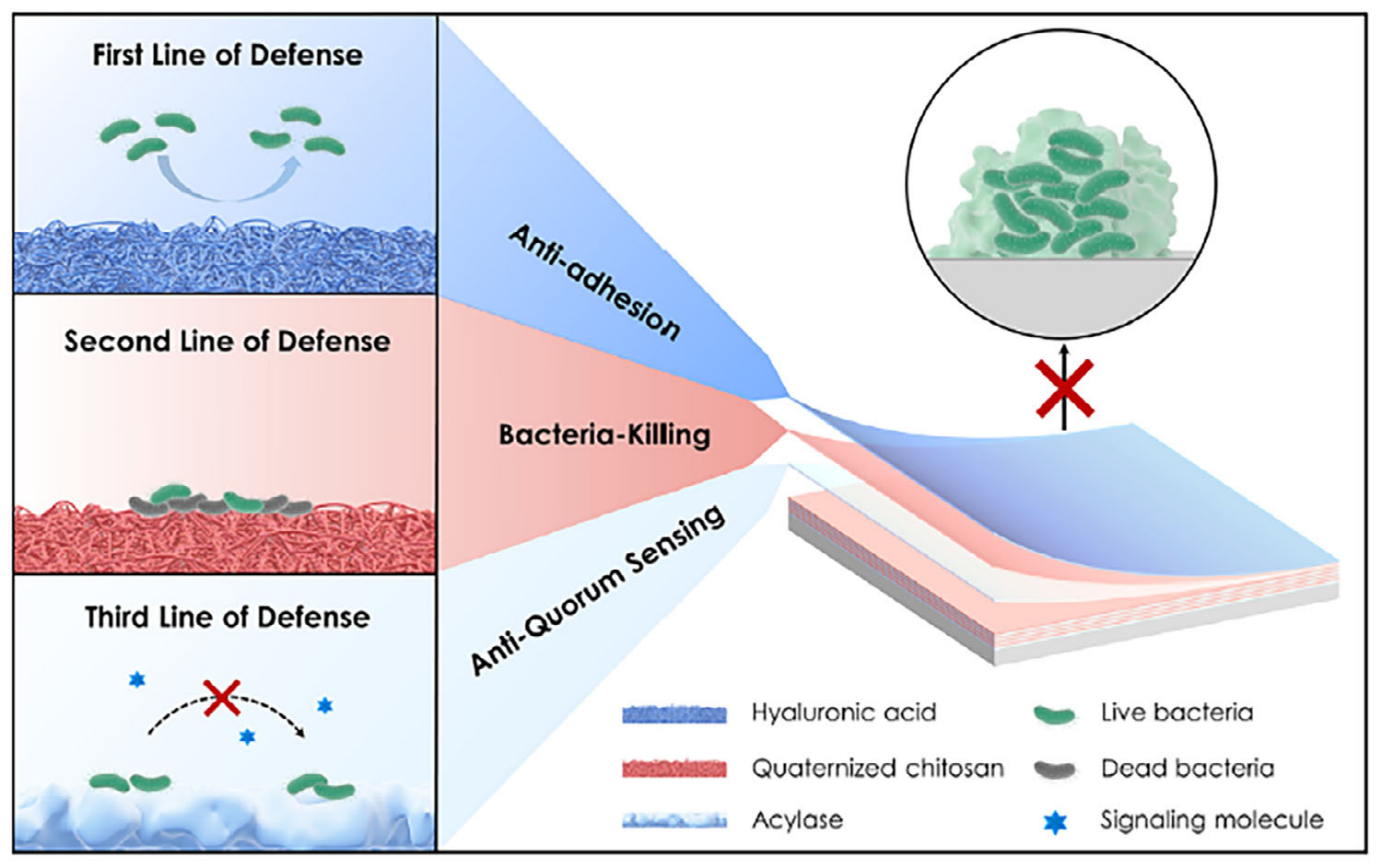
A multilayered antimicrobial coating composed of a degradable antiadhesive layer followed by a contact killing with the release of antiquorum sensing molecules (Reproduced from [59], Copyright 2022, with permission from Elsevier).

#### 2.2.3 Light‐Activated Systems

Light‐activated antimicrobial coatings represent a novel class that enables non‐releasing, on demand antimicrobial activity through externally triggered photochemical processes. In these systems, materials such as titanium dioxide (TiO_2_), up‐conversion particles or other photoactive additives generate reactive oxygen species (ROS) or emit ultraviolet (UV) radiation upon illumination, enabling a non‐releasing, surface‐bound mechanism of microbial inactivation. Unlike releasing systems, antimicrobial function does not rely on continuous diffusion of active ingredients and is therefore less prone to depletion over time. Furthermore, because the activity is surface‐bound and externally controlled, light‐activated systems offer the potential to reduce environmental and health concerns associated with metal ion release.

From a high touch surface perspective, light activated strategies are particularly attractive because antimicrobial activity can be triggered when needed, rather than being continuously active. This contrasts with contact‐killing surfaces, which require direct microbial contact and with release‐based systems, which may lead to uncontrolled exposure to active components. However, light‐activated systems also present inherent limitations. Their antimicrobial efficacy is strongly dependent on light availability, which can restrict performance in shaded or indoor environments unless supplemented by artificial illumination. Recent developments focus on overcoming the limitations of classical photocatalytic coatings, especially their dependence on UV light, by enabling visible light activation and improved compatibility with practical coating formulations, positioning these systems as next generation antimicrobial technologies.

Incorporation of up‐conversion or photocatalytic particles into coating matrices requires careful optimization to balance optical transparency, mechanical integrity, long‐term stability or gas and water permeability. Importantly, currently available antimicrobial testing standards do not adequately account for controlled illumination conditions, making performance assessment and comparison challenging. The antimicrobial activity of light‐activated coatings is governed by distinct photophysical and photochemical mechanisms, which are briefly outlined below to highlight their relevance for antimicrobial surface design. One common photoluminescence process is down‐conversion, in which the excitation light has a shorter wavelength (higher energy) than the emitted light [60]. A well‐known example is LED lighting, where blue light emitted by a semiconductor chip excites phosphors that convert it into lower‐energy visible light, producing white illumination. In contrast, up‐conversion mechanisms involve absorption of lower‐energy light and emission of higher‐energy radiation, such as UV light. Up‐conversion processes from visible to UV light in inorganic particles have been extensively studied. The mechanisms include energy transfer in lanthanide‐doped phosphors, two‐photon absorption in nanocrystals and quantum dots, triplet‐triplet annihilation, photon avalanche effects, defect‐related emissions in materials such as ZnO and TiO_2_, and co‐doping strategies that enhance energy transfer efficiency [61, 62, 63, 64]. Figure 4 illustrates respectively the up‐conversion mechanism by energy transfer up‐conversion (4a), as well as an example emission spectrum of such up‐converters after excitation with 460 nm (4b). Overlapping of the emission spectrum of up‐converting materials with germicidal absorption curves known from literature (4c) indicates that inactivation of microorganisms can occur in the UVC/UVB range after excitation with visible light. Nanostructuring further improves up‐conversion efficiency by increasing surface area and inducing quantum confinement effects. The UV radiation emitted by up‐converter particles can induce photodimerization at dipyrimidine sites in microbial DNA, interfering with replication and transcription and inducing oxidative DNA damage, including single‐strand breaks [65, 66]. Importantly, the emission spectra of certain up‐converter materials overlap with UV absorption ranges associated with microbial inactivation mechanisms, as defined in DIN 5031‐10, supporting their antimicrobial potential [67]. For instance, Cates et al. [68] developed antimicrobial surfaces based on Pr^3^^+^‐doped Y_2_SiO_5_. By incorporating Li and Gd co‐dopants, UVC emission was enhanced by up to 14‐fold, resulting in inhibition of *P. aeruginosa* biofilm formation and an approximately 1.01 log reduction when using 50 µL of 3 × 10^7^ of *Bacillus subtilis* spores under standard indoor fluorescent lighting [68].

**FIGURE 4:**
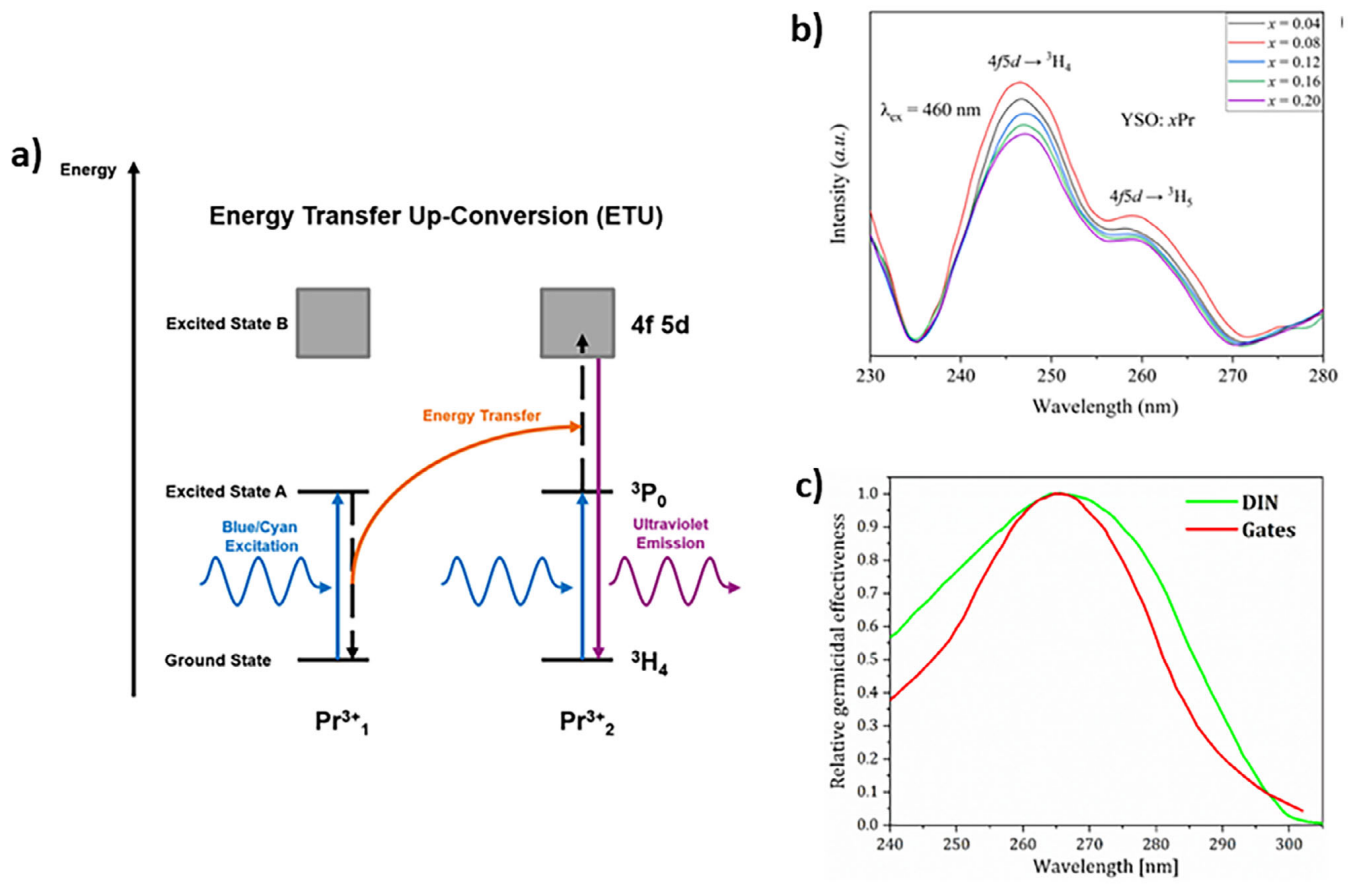
(a) Schematic illustration of the energy transfer up‐conversion mechanism: a 488 nm photon excites the ion from the ground state ^3^H_4_ into an intermediate excited state ^3^P_0_. A second photon is absorbed before relaxation occurs, promoting the ion to a higher excited state within 5d band, from which ultraviolet emission can occur via radiative relaxation. (b) Emission spectrum of up‐converter with excitation at 460 nm (Adapted from [69], Copyright 2022, Springer Nature), and (c) Added absorption spectra of bactericidal effectiveness by two different models (Adapted from [70], Copyright  under the terms of the Creative Commons CC‐BY), overlapping with emission spectrum of 4b [67].

Another widely investigated light‐activated antimicrobial mechanism is photocatalysis. When a semiconductor absorbs photons with energy equal to or higher than its band gap, electrons are promoted from the valance band to the conductive band, leaving positively charged holes. These electron‐hole pairs must separate and migrate to the surface, rather than recombine internally, to drive redox reactions [71]. Charge separation is favored by high crystallinity (fever defect related recombination centers), small grain size (shorter diffusion paths to the surface) and exposed reactive crystalline planes such as anatase (001) [72]. The reaction rates also depends on availability of surface adsorption sites and therefore on particle size and surface area [73, 74]. The anatase phase of TiO_2_ is the most extensively studied photocatalysts [75]. The photocatalytic activity also depends on phase composition, with the ration of anatase to rutile influencing the performance [76]. Owing to its wide band gap (∼3.2 eV for anatase and ∼3.0 eV for rutile, corresponding to threshold wavelengths of ∼388 nm and ∼413 nm), pristine TiO_2_, is activated only by UV. However, the white LED light typical of indoor lighting emits almost no UV. Visible/LED activation therefore requires band gap modification: doping variants (e.g. N‐, S‐, or metal‐doped TiO_2_) [77] or oxygen vacancy defects [72] extend activity to visible/LED range relevant to indoor high touch surfaces.

At the surface, the hole and electron react with adsorbed water and oxygen to form ROS, mainly hydroxyl radicals and superoxides, that inactivates microorganisms via membrane, protein and nucleic acid damage, in addition to mineralizing organic compounds (Figure 5) [77, 78, 79]. ROS yields, and thus its antimicrobial efficacy, also depend on photon flux, humidity, and surface cleanliness which on high touch surfaces often differ from standard laboratory conditions: laboratory conditions may not predict performance on high touch surfaces [80]. Regarding photon flux, there is no intensity threshold: every absorbed photon of sufficient energy can generate an electron‐hole pair that may initiate a surface reaction if it does not recombine first [81]. At very low light intensities, however, ROS may be generated in quantities too small to produce a relevant antimicrobial effect. Since both the intensity and the spectral overlap between light source and photocatalyst determine the effective photon dose, studies should report the light source type, emission spectrum or peak wavelength, irradiance at the sample surface (e.g., mW/cm^2^), distance, and exposure time or resulting dose (J/cm^2^). Since the determination of absolute irradiance values involves many additional parameters, such as surface reflectance, fluctuations in light intensity, or differences in radiation incoupling due to variations in coating thickness, specifying a required dose remains subject to considerable uncertainty. Surface conditions also matter: organic soiling has been reported to affect performance more than light intensity or distance [82], and humidity plays a similarly important role [83].

**FIGURE 5:**
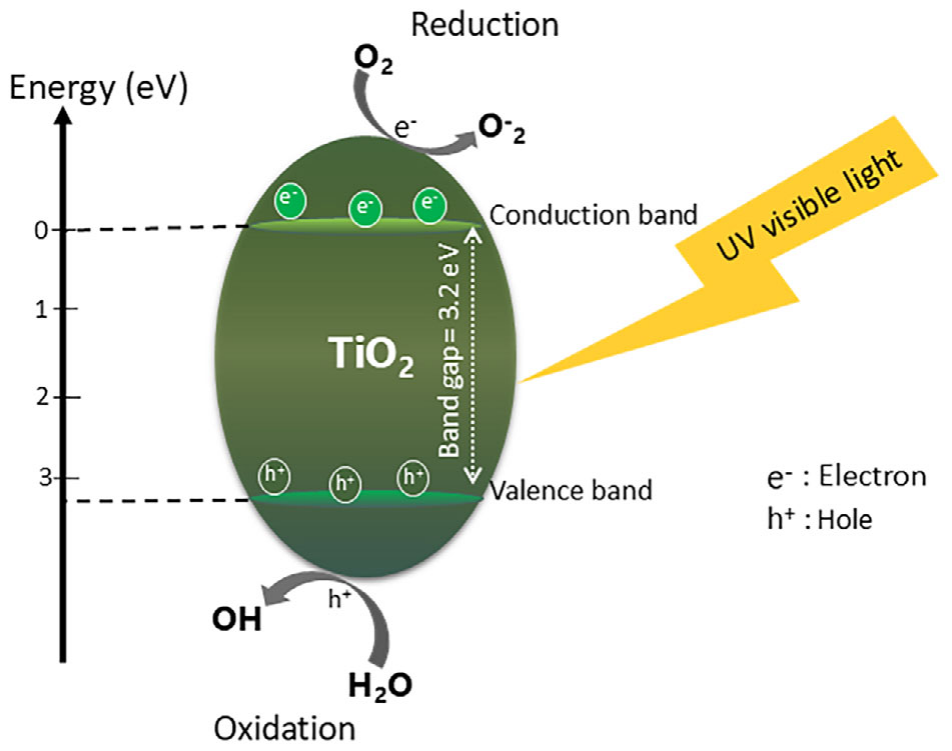
Schematic illustration of photocatalytic processes. Upon light irritation with energy ≥3.2 eV band gap, TiO_2_ generates electron‐hole pairs that reacts with oxygen and water to form reactive oxygen species (Adapted from [94], Copyright 2022, with permission from Elsevier).

Several studies have demonstrated the applicability of TiO_2_‐based coatings for high touch surface scenarios. For example, Fu et al. [84]. and Won et al. [85]. developed highly transparent nano‐TiO_2_ coatings applied to glass substrates with high surface coverage for touch screens of medical devices. Anatase‐rutile composite coatings achieved approximately 3.65 log reduction against MS2 bacteriophages after 90 min of UV illumination [84] and showed higher than 80% reduction (∼0.7 log) of attached *E. coli* after 45 min (inoculum: 3 mL at ∼1 × 10^8^ CFU mL^−1^) [85]. However, pristine TiO_2_ photocatalysis is predominantly UV‐driven, limiting performance under typical indoor lighting conditions. Therefore, efforts have focused on material modification strategies such as doping, surface engineering, and hybridization to enable visible‐light activation and enhance photocatalytic efficiency [86, 87]. For instance, Krumdieck et al. [88]. reported a nanostructured TiO_2_ coating composed of anatase and rutile, providing high surface area and incorporating amorphous carbon. This architecture enabled antimicrobial activity under visible light (1500 lumens, representative of typical healthcare lighting), achieving more than a 3‐log reduction in *E. coli* (inoculum: 50 µL at ∼1 × 10^7^ CFU mL^−1^) after 4 h of illumination without elemental doping [88]. The authors further demonstrated the suitability of this coating for large‐scale industrial application on high touch surfaces in hospital environments [88].

Organic photosensitizers exhibit strong absorption bands across different regions of the visible light spectrum, enabling antimicrobial activity through the generation of ROS. Compared with biocides, organic photosensitizers can exhibit antimicrobial effects at lower concentrations and are active against a broad variety of microorganisms including bacteria, viruses, and fungi [89, 90]. The breadth of activity of photosensitizers is particularly relevant for high touch surfaces, which are typically contaminated by a mixed microbial burden rather than a single pathogen type; a coating effective only against bacteria, for instance, leaves viral or fungal contamination on the same surface unaddressed. Their physicochemical and photophysical properties can be tuned to modulate absorption wavelength, solubility, and the quantum yields of singlet oxygen production [89]. Furthermore, they can be designed to be cationic, anionic or neutral depending on targeted electrostatic interactions [89]. For instance, cationic organic photosensitizers may exhibit enhanced activity against Gram negative bacteria; their positive charges displaces the divalent cations (Ca^2+^, Mn^2+^) that cross‐bridge and stabilize the negatively charged liposaccharide layer of the outer membrane, disturbing this barrier and promoting photosensitizer uptake, a pathway is not available to Gram positive bacteria, which lacks an outer membrane [91]. Photosensitizers can also be embedded in or covalently incorporated into surface coatings [90]. Upon light exposure, these coatings activate the photosensitizers, leading to ROS generation and the formation of the self‐disinfectant surfaces. Unlike release‐based or contact‐killing strategies, photosensitizer‐containing coatings can inactivate pathogens without requiring leaching of the active agents or direct penetration into cells [90]. Instead, singlet oxygen generated near the microbial membrane can diffuse into the cell and damage cellular components. Eichner et al. [92]. investigated the efficacy of photosensitizer‐containing coatings in reducing bacterial contamination in near‐patient hospital environments under real‐life conditions. The coatings achieved an approximately 4‐log reduction against several human pathogens under laboratory conditions, while near‐patient areas treated with photodynamic coatings exhibited approximately three‐fold lower mean bacterial contamination levels compared with untreated areas [92]. Similarly, another study reported a significant reduction in bacterial contamination on surfaces in urban buses under real‐life operating conditions [93].

Despite their advantages, light‐activated antimicrobial systems raise specific safety and regulatory considerations. Uncontrolled generation of ROS at high concentrations has been associated with potential tissue damage and genotoxic effects. From a regulatory standpoint, ROS generated in situ is classified as biocidal agent under the Biocidal Products Regulation, whereas up‐conversion‐based systems may fall outside of this framework due to their predominantly physical mode of action (the EU Biocidal Products Regulation [95]).

### 2.3 Hybrid Systems

Hybrid antimicrobial coatings combine two or more antimicrobial concepts within a single coating system. For high touch surfaces, which are exposed to repeated contamination, cleaning, and mechanical wear, relying on a single antimicrobial mechanism is often insufficient to maintain long‐term performance. Hybrid strategies are therefore being explored to compensate for the weaknesses of individual approaches, for example by combining fast surface inactivation with longer‐lasting protection or by reducing fouling and depletion effects. Recent studies show that integrating multiple functions within one coating can improve robustness and applicability, making hybrid systems a promising direction for next generation antimicrobial coatings on high touch surfaces [96, 97].

For instance, Lu et al. [98]. developed a hybrid antimicrobial coating that combines antifouling and release‐based strategies for transparent and durable high touch surface applications. The coating is based on a nanosilica resin matrix composed of lithium and potassium silicates, silver nitrate, and N‐alkylpropylaminoglycine. The system achieved approximately 99% bacterial population reduction (2‐log) against *E. coli* and *S. aureus* (both bacterial inoculum: 150 µL at 1 × 10^9^ CFU mL^−1^). Importantly, the coating maintained its hydrophilicity and structural integrity, with efficacy against *E. coli* and *S. aureus* unaffected, after 2000 friction cycles of manual a cleaning cloth abrasion (applied equipment and load not reported), demonstrating suitability for repeated‐use environments [98]. Another example of a hybrid approach was reported by Manuoras et al. [99], who combined contact‐active and light‐activated antimicrobial strategies within a single coating system. The coating integrates quaternized chitosan as a contact‐killing component, an acetal‐based crosslinker providing pH‐responsive, self‐cleaning behavior, and TiO_2_ nanoparticles with reduced graphene oxide to enable visible‐light activation. This multifunctional design allowed antimicrobial activity both in the dark, achieving 2–3 log reduction, and enhanced performance under visible‐light illumination, reaching 5–6 log reduction against *E. coli* and *Bacillus cereus* (inoculum: 100 µL at 1 × 10^8^) [99]. Fu et al. [84]. combined release‐based and light‐activated strategies to produce coatings for high touch surfaces using Ag and TiO_2_ nanoparticles (Figure 6). Under UVA‐LED irritation, the addition of Ag nanoparticles enhanced the antiviral performance against bacteriophage MS2, achieving 5–6 log reduction within 90 mins of light exposure (inoculum: 4.2 mL at ∼1 × 10^6^ PFU mL^−1^) [84]. While the coating showed limited antiviral activity under dark conditions [84], similar formulations exhibited strong antibacterial activity against *E. coli*, highlighting the complementary roles of release‐based and photoactivated mechanisms [85].

**FIGURE 6:**
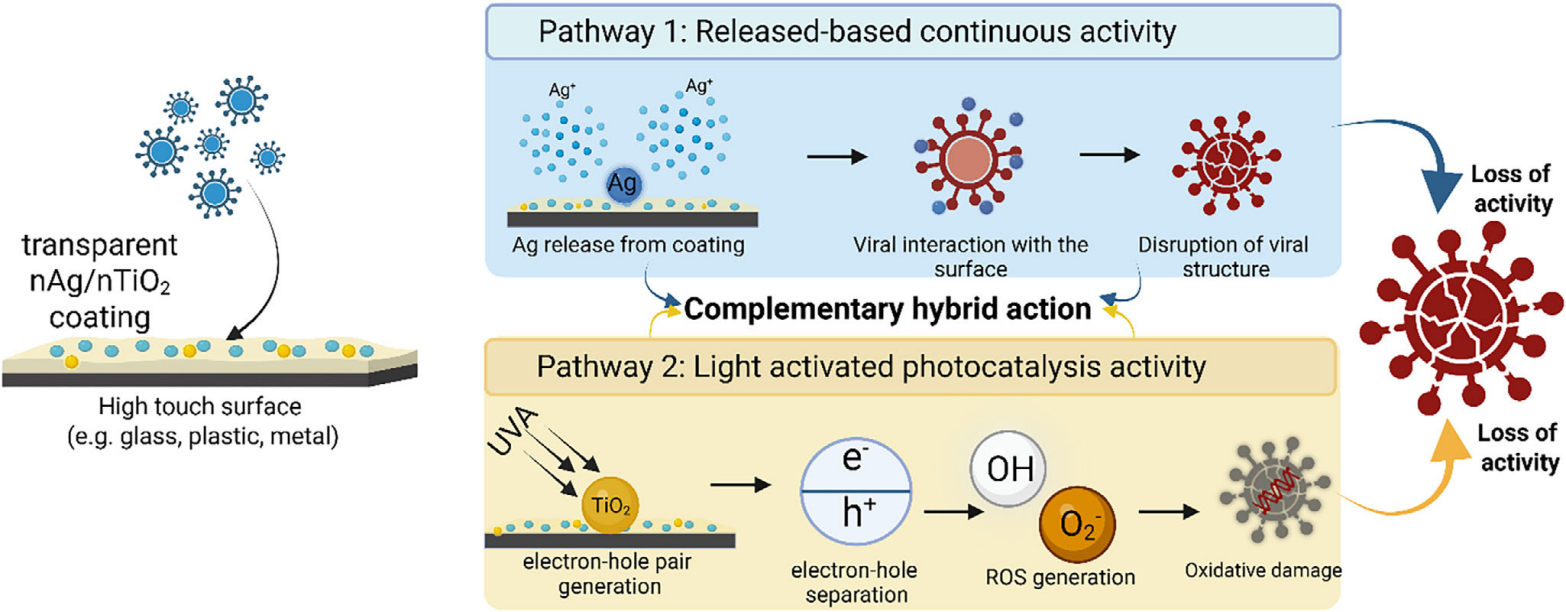
Proposed antimicrobial mechanism of nAg/nTiO_2_ hybrid photocatalytic coating. The schematic illustrates the complementary contributions of silver associated antiviral activity and light activated TiO_2_ photocatalysis, inspired by the findings reported by Fu et al. [84] Created in BioRender. Mutlu, N. (2026) https://BioRender.com/f16ber8.

## 3 Real‐Life Relevant Antimicrobial Testing Approaches

Surface mediated transmission via hand contact represents a critical route for microbial spread. However, the identification of promising candidates remains strongly dependent on how antimicrobial efficacy is assessed. For high touch surfaces in particular, antimicrobial performance is highly affected by environmental conditions, contamination modes, and usage patterns, which often differ substantially from those applied in common standardized laboratory tests. In addition, as the antimicrobial strategies discussed in the previous sections rely on fundamentally different modes of action, their performance cannot be fully compared or translated to real‐world use without testing approaches that reflect realistic exposure scenarios [100, 101]. The following section therefore discusses key limitations of current standardized methods and highlights emerging testing strategies designed to better simulate conditions relevant to high touch surfaces.

The development of novel antimicrobial materials and coatings relies upon a thorough, accurate, and reproducible efficacy testing process via standardized testing methods [102]. Currently, common standardized methods (such as ISO 22196:2011 [103]) assess the efficacy of candidate coatings and surfaces using environmental conditions (temperature, relative humidity, inoculum volume etc.) that are not reflective of the conditions that the material will be subjected to once in use. These in‐use conditions have a profound impact on the way in which most antimicrobial materials behave and perform in practice [102]. While many of the current standards have utility for screening candidate materials for activity and quality control processes, the development of new test methods that evaluate efficacy under more realistic conditions is of the utmost urgency to allow the action of antimicrobial surfaces and finishes to be assessed for function in practice.

As the environmental conditions in many end‐use cases vary so vastly even in the same location [104], it is difficult to define a set of conditions that can accurately be employed when determining whether a material can be considered antimicrobial. For example, hospital regulations often require a relative humidity of between 40%–60% [105], while the conditions surrounding mobile phones will depend on the current temperature and relative humidity of the outdoor environment [106], which can quickly change when a person enters an indoor, climatized building. This is further complicated by the often greatly varied climates of different countries, such as the low relative humidity often present in desert regions [107] compared to tropical regions [108]. To accommodate these changing conditions, it is important to either test candidate materials in a worst‐case scenario so there can be confidence in the material overall or to test the material based on a specific end‐use scenario and only approve the use of the material for that case. Likewise, there is no single target reduction that confirms efficacy; instead, a suitable reduction should be decided based on the intended application. For example, a coating in hospital intensive care unit would benefit from a large reduction in microbial population, whereas for non‐critical applications a 2 or 3 log reduction may suffice. A reduction to below the limit of detection, while the untreated control still yields recoverable organisms, is the strongest result a test can show, although its magnitude depends on the inoculum applied. Additionally, many current standardized test methods apply an unrealistic inoculum to the surface, often adding hundreds of microliters to the surface when this likely would not be the case in practice. For example, an end‐use where coughing is one of the primary mechanisms of microbial transfer via fomites [109] results in the release of individual contaminated droplets that are much smaller than the applied inoculum, often only 1–5 microliters in size [110]. Similarly, transfer via hand contact (or gloved hand contact) often transfers almost no liquid to the surface. The development of antibacterial surface test methods should aim to apply bacteria in a low moisture manner (e.g. by low inoculum volume or simulated touch transfer) as well as altering the environmental conditions during the contact period to that more analogous to a typical end‐use environment. Additional consideration should be given to repeat inoculation/reuse of a material, durability assessment, and the long‐term impact of cleaning practices on the efficacy of the material.

Recently, efforts have been made to produce novel methods and standards that are able to better simulate end‐use. For example, ISO 7581 [111] enables the user to assess antibacterial efficacy of a surface under non‐submerged conditions by inoculating microorganisms within a 1 µL droplet, then spreading it and allowing it to dry, followed by incubation within a moderate relative humidity (35%–60%) for 1 and 24 h prior to recovery. While this offers advances over ISO 22196:2011 [103], this method may still introduce variability as the spreading action of the droplet can vary intra‐laboratory and inter‐laboratory [112]. Multiple variations of this small droplet method exist in the literature in terms of in inoculum volume, relative humidity, and species tested [113, 114, 115]. Other examples of more realistic methods include dry deposition (non‐suspended inoculum) using a 3D printed apparatus [116] which can simulate touch transfer of bacteria in a more reproducible manner than current methodologies, especially as no current standard method exists for this case and other methods described in the literature often use finger coverings that will provide variability in the pressure applied and contact area used [117]. While no standards are currently available for touch transfer of bacteria, ISO/WD 25299 evaluates the viral hand touch contamination of surfaces, but still relies upon difficult to reproduce methodology such as using a finger to transfer [118]. Methods that utilize aerosol deposition of microorganisms capable of reproducibly mimicking a splash, sneeze or cough have recently also been developed [119]. Novel methods that also account for light activation (e.g. for photocatalytic coatings) are further challenged by the incorporation and standardization of an appropriate light source on top of the requirement for realistic deposition.

The important consideration for test methods that intend to be more than either a basic proof of principle or that demonstrate the potential of a material to act against environmental microbial contamination, is that they reflect the action that can be anticipated in practice. For example, the strains selected for standardized testing methods are chosen for being well characterized, phenotypically predictable and widely available. However, these strains are unlikely to be as relevant to end‐use scenarios where antimicrobial and desiccation resistance are continually selected for and are likely to cause a higher number of infections in the built environment. Similarly, when assessing antiviral performance, bacteriophage (viruses that infect bacteria) are often used as a surrogate for human viruses with similar structures (enveloped or non‐enveloped) as they are generally safe to use, cost‐efficient and do not require the upkeep of complex and costly human host cell lines to perform propagation. Bacteriophage such as MS2 (non‐enveloped human norovirus surrogate) and Φ6 (enveloped human coronavirus surrogate) are often used and often provide reliable data on antiviral performance.

This balance of analogy to end‐use conditions and high repeatability, ease of use, and requiring large differences between control and candidate surfaces is an ongoing challenge in standards development. An example of this is that bioburden present in clinical environments, public transport, and homes is both significantly smaller than employed in standardized tests and very heterogenous in its distribution. This highlights the difficulty and expense in using the exact conditions expected in service, which would likely require complex, time‐ and resource‐consuming field trials that are impractical on larger scales. Therefore, it would be advantageous to measure the performance against claims made for a technology under the conditions most closely resembling what it will experience in use. This would provide scientifically robust support for these claims and provide confidence in benefits from its use (and that may be detectable over time in outcomes such as a reduction in hospital acquired infections, etc.). In addition, such methods would satisfy the need for robust data that supports claims made in many international regulatory regimes (e.g., the EU Biocidal Products Regulation [95]) that many current methods do not.

## 4 Human and Environmental Safety Considerations

Antimicrobial coatings can pose potential risks to both human health and the environment. Therefore, the development of new coatings should not only be guided by antimicrobial efficacy but must also be supported by a rigorous safety assessment [120]. The widespread and often continuous use of these coatings on consumer products, medical devices, and in built environments necessitates a comprehensive evaluation of their short‐ and long‐term impacts on humans and ecosystems, considering realistic exposure scenarios, material durability, degradation pathways, and end‐of‐life behavior [7]. Reflecting these concerns, antimicrobial coatings are subject to increasing regulatory oversight. In the European Union, coatings that rely on the release of biocidal agents are regulated under the Biocidal Products Regulation (the EU Biocidal Products Regulation [95]), with their chemical components additionally governed by REACH, while in the United States they fall under EPA regulation via FIFRA, with stricter requirements for products making public health claims. Collectively, these regulatory frameworks emphasize life‐cycle risk assessment, controlled release of active components, and minimization of environmental emissions.

### 4.1 Release Mechanisms

In the context of human and environmental considerations, understanding how, under what conditions, and in which form, the active components are released from the antimicrobial coating is critical. Release directly links to the coating mechanism of action, long‐term durability and efficacy, and realistic exposure of users and environmental emission during the coatings’ life cycle [7, 121]. Many antimicrobial coatings are nano‐enabled or contain particulate active components (e.g. metal or metal oxide micro‐ or nanoparticles) and can therefore be regarded as a specific class of nanocomposites. As a systematic review of the general release mechanisms for antimicrobial surface coatings, particularly high touch applications, is still lacking, the concepts from related fields are adapted here to provide a mechanistic framework for release assessment.

From a physicochemical perspective, active components can be released from antimicrobial coatings via four main mechanisms: diffusion, dissolution, desorption and matrix erosion (Figure 7) [122, 123, 124]. These mechanisms often occur simultaneously, and their relative contributions depend on the nature of the active component, the coating matrix and the use conditions [122]. Depending on coating chemistry and stressors, released materials may occur as dissolved species, particulate matter (either free or matrix embedded), and/or transformed products; thus, release mechanism and form are closely linked for exposure assessment.

**FIGURE 7:**
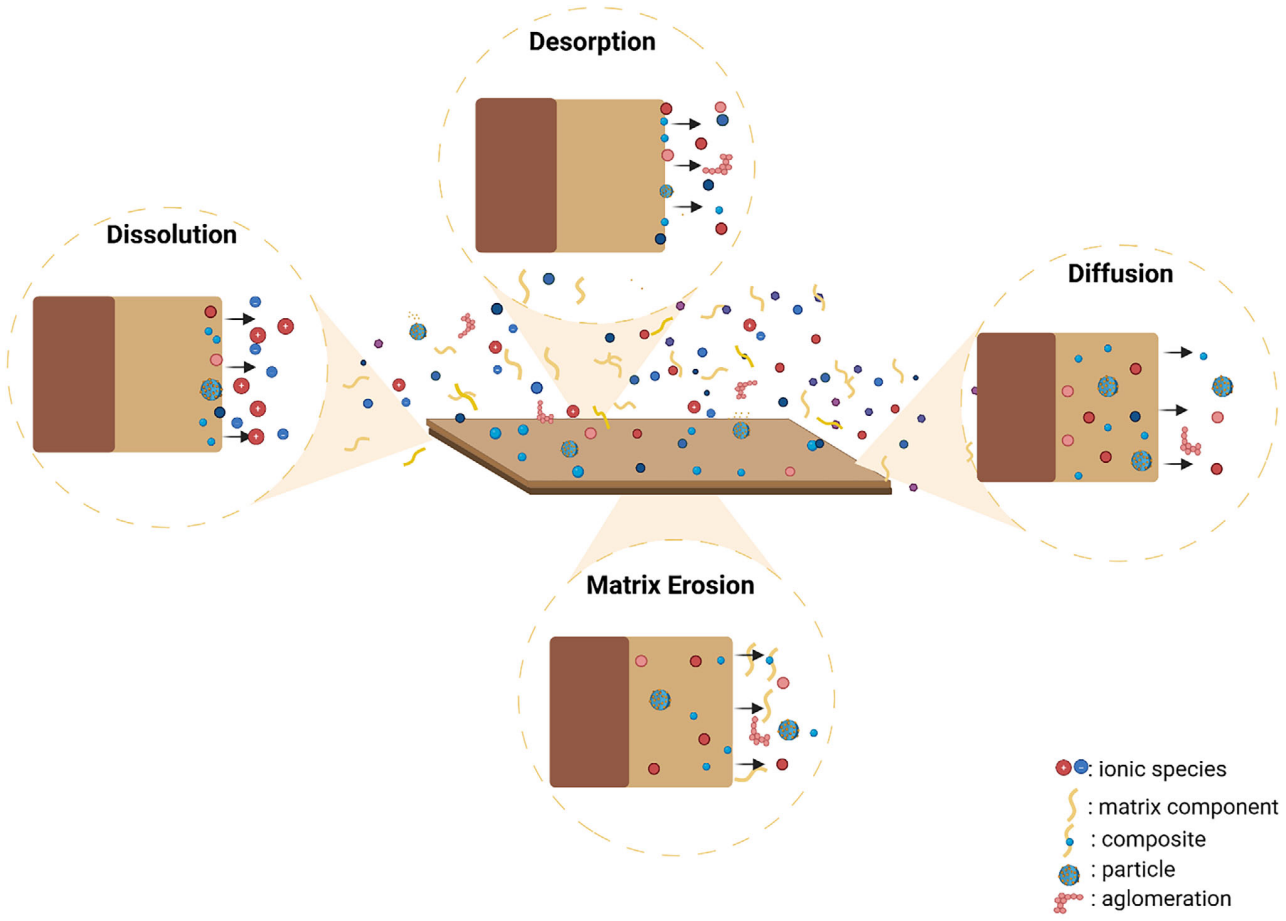
Schematic illustration of possible release mechanisms from antimicrobial coatings include i) desorption of loosely bound particles from the surface, ii) diffusing driven by a concentration gradient, iii) dissolution of particles from the coating into surrounding medium as ions, and iv) degradation of the coating matrix, resulting in the release of particles previously immobilized within the matrix. (Created in BioRender. Mutlu, N. (2026) https://BioRender.com/fy8lo2c, adapted from [124], Copyright 2014, with permission from John Wiley and Sons).

Dissolution is the key mechanism for many antimicrobial coatings, particularly those that rely on the controlled release of soluble antimicrobial agents. For metallic silver, for example, the primary antimicrobial effect is attributed to the sustained release of Ag^+^ ions generated by the oxidation of metallic silver in an aqueous environment. Silver‐based antimicrobial coatings are by far the most widely studied and applied, followed by systems based on titanium, copper, zinc, chitosan and quaternary ammonium compounds. The literature indicates that silver, copper and zinc are frequently released as ionic species, whereas chitosan and titanium are employed as carriers [7, 120].

Diffusion refers to the migration of the active component from the bulk of the matrix (often a polymer) to the contacting media, driven by a concentration gradient [122]. For instance, diffusion‐driven Ag release from Ag^+^‐loaded mesoporous silica particles intended for high touch surfaces in healthcare settings can result in low but sustained ion release, as Ag^+^ gradually diffuses from the nanopores into the surrounding medium [53].

In the desorption mechanism, weakly bound or adsorbed active components on the surface of the coating (e.g. loosely attached nanoparticles) partition into the surrounding medium [122, 124]. Diffusion is inherently hindered for large molecules and particles, particularly in dense or highly crosslinked matrices, making dissolution and desorption more dominant under such conditions [122, 124]. In diffusion and desorption, released materials may remain in particulate form, although their morphology, composition, or surface characteristics may alter during or after the release process [122]. Matrix erosion is a dynamic release process and involves the relocation of the active components from the coating due to chemical (e.g. UV‐assisted, thermal or hydrolytic degradation) or physical destruction (e.g. mechanical abrasion) of the matrix component, thereby increasing the active component release. For high touch surfaces, mechanical erosion is an especially relevant release mechanism, as the coatings are exposed to frequent hand contact, cleaning, and disinfections [123]. Indeed, recent studies have recognized the need to focus on the release and transformation of particles from various surfaces via dermal contact [125, 126, 127, 128, 129, 130]. For instance, Clar et al. [127]. demonstrated that zinc and copper containing coatings can release transformed species during simulated dermal contact, highlighting that users may be exposed to chemical forms with different toxicological profiles than the pristine particles [127].

Importantly, the different release mechanisms are not separated from each other. Diffusion may follow initial desorption; dissolution is inherently coupled to diffusion and matrix erosion can influence all other processes by altering the matrix or particle properties [124]. In complex structures, such as core‐shell particles, individual components may dissolve or detach at different rates [124]. Overall, the interplay between dissolution, diffusion, desorption and matrix erosion determines the profile and amount of active component release from antimicrobial coatings.

A critical challenge is that linking observed release to specific mechanisms and importantly to the released form remains difficult with current characterization techniques, which often rely mainly on elemental analysis. Distinguishing whole particles from dissolved ions therefore is a significant challenge. As released particles may have different toxicological profiles and environmental impacts than the pristine materials [131, 132], determining the form of released material is a key property in release studies. For nano‐enabled polymer composites similar to those used in particle‐based antimicrobial coatings, the most frequently identified particles are those of the matrix alone, and slightly less often, matrix particles containing the nanomaterial partially or fully embedded; far less frequently one can observe that the added nanomaterial is entirely dissociated from the matrix and present in free form [133]. Because release‐based antimicrobial systems represent the first generation of antimicrobial coatings, dissolved ionic species are expected to be one of the prominent forms of the released active species [7]. Characterizing the pristine active ingredients and their distribution within the composite together with a detailed assessment of the released materials will provide a basis for deriving structure‐function relationships [134]. Such mechanistic insights can form the basis for the subsequent assessment of environmental exposure and effects, as well as for the development of predictive frameworks for safe and sustainable by design strategies for antimicrobial coatings [134, 135].

Studies on particle release from nanocomposites indicate that matrix properties, composite architecture, particle embedding, and applied mechanical forces strongly influence release kinetics, particle size and number, and release form [7, 122, 124, 131, 134]. Nanoparticles embedded in solid matrices are expected to be released gradually and only partially over a product's life [131]. From an environmental and consumer safety perspective, maintaining strong particle‐matrix interactions and stability of the coating formulation is therefore a critical aspect to minimizing exposure: coatings with lower stability may release active components unintendedly during regular use, cleaning and disinfection or disposal, increasing both human and environmental exposure [136, 137, 138].

Antimicrobial coatings may face numerous stressors that influence durability and performance, including aging, UV degradation, weathering, detergent cleaning, oxidation, washing cycles, and sustained mechanical abrasion [139]. Such impacts may reduce antimicrobial efficacy or cause partial detachment from the substrate [136, 139]. To evaluate mechanical stability, abrasion resistance can be measured using e.g. a Nu‑Martindale Abrasion and Pilling Tester, allowing comparison between coated and uncoated control substrates [136]. Additional durability assessments can be performed with instruments certified under EN ISO 7854 [140], simulating flexing, folding, and material fatigue typical of packaging, transport, and daily use conditions [138]. Testing method PAS 2424:2014 is a relevant example, combining mechanical abrasion with microbial recontamination to assess disinfectant residual efficacy over 24 h [141]. However, it does not apply to permanently bound coatings, and its limited abrasion regime does not adequately reflect high touch surfaces contacted multiple times daily, suggesting contact frequency adaptations of this approach could improve coating efficacy testing. These standardized tests help predict whether coatings will remain functional under realistic wear and tear conditions. Many novel coatings, especially those incorporating nanoparticles, hybrid composites, or responsive release systems, require adapted or complementary evaluation approaches compared to single chemical entities. Given these complexities, a modernized and more holistic testing framework is necessary to ensure that antimicrobial coatings perform effectively while maintaining human and environmental safety throughout their entire lifecycle.

### 4.2 Human Safety Considerations

Antimicrobial coatings are frequently applied in environments where humans experience close, repeated, or even continuous contact with treated surfaces [142, 143]. Additionally, if adequate workplace hygiene is not enforced, exposure may also occur during the production of antimicrobial coatings. Because these coatings are intended for use in the immediate proximity of people, or even designed for direct touch, their safety assessment should reflect realistic exposure routes [144]. Skin contact represents the most probable scenario during use, as antimicrobial agents may potentially be released through sweat and can be transferred to the skin through the mechanisms discussed above [145]. This is especially relevant when individuals sit, touch, or lie on coated materials for prolonged periods, which may even allow transfer through several layers of textile fabric [136, 145]. Therefore, a key strategy to guide the development of novel antimicrobial coatings for high‐touch surfaces is to determine, at an early stage, which regulated product category the coating might eventually fall into. If a medical device is considered, the ISO 10993 series (standards for the biological evaluation of medical devices) recommends suitable tests to assess certain biological effects. Such biological effects are derived from the outcome of a prior biological risk analysis [146, 147]. If nanomaterials are part of a next generation antimicrobial coating of a medical device, further guidance is given in ISO/TR 10993‐22, “Guidance on nanomaterials” [148]. This technical report summarizes the latest information for nanomaterials in medical devices and is currently under review by the ISO committee (ISO TC 194 WG 17).

Typical biological effects for the evaluation of touch surface coatings might be determined by a “short‐term exposure” (total exposure time ≤ 1 day) and a “contact to intact skin”, and might then be: cytotoxicity, skin sensitization, and skin irritation [149, 150, 151, 152]. For these biological effects, it must be decided if sufficient testing data are already available in the literature to properly conduct a biological risk assessment or if additional biological effects might need to be evaluated. Further, all described safety tests used to gain data on the biological effects of finished medical devices within the ISO 10993 series might already be of interest in the design and development phase of such products.

Over the past decade, human cell‐based in vitro toxicity assays have advanced significantly and are now widely used for development‐phase hazard identification, compound screening, and pathway‑based toxicity assessment, often paired with predictive computational models [153]. On one hand, simple cell‐based toxicity assays which are accepted by regulatory bodies have a high readiness level. On the other hand, complex multi cell‐based toxicity assays are more clinically relevant and more predictive than the simpler cell‐based assays (as complex as needed, as standardized as possible). More broadly, the field of toxicology is shifting from traditional endpoint‐based approaches to more cell‐based and mechanistic toxicology, where new approach methodologies (NAMs) become more relevant, replacing animal testing with in vitro, *in chemico*, and in silico methods. Studying antimicrobial coatings presents unique challenges as most regulatory toxicity guidelines were originally developed for soluble chemicals, but more and more include the assessment of solid, immobilized coatings which may contain embedded antimicrobial active compounds [154, 155]. To address this gap, the standardized KeratinoSens assay, described in the 442D OECD Test Guideline [154], which is used to measure skin sensitization by chemicals and is suggested as one option for testing skin sensitization by medical devices, as discussed in ISO 10993‐10, Annex C [151], has been adapted for use with artificial sweat extracts of antimicrobial coatings following ISO 10993 principles [136, 154]. This adaptation allows for a more realistic simulation of skin exposure scenarios and enables rapid screening due to its high throughput [154].

To assess cytotoxicity, ISO 10993‐5 outlines suitable in vitro test protocols [152]. These test protocols involve extract and direct contact tests for medical devices. Here, however, a challenge is posed by the fact that no assay is described for the quantitative analysis of cytotoxicity in the cell‐material direct test. Only a qualitative analysis is available at this point, complicating interpretation. Having a quantitative test option would make sense in the case of developing next‐generation coatings for frequently touched surfaces, and is being worked on, for example, by the NOVA initiative (www.eu‐nova.eu).

A cell culture tool designed to investigate direct cell‐material interactions in the form of cell‑based assays with a quantitative read‐out is the so‐called ClicKit‑Well system [156], which enables testing of solid, opaque, or textile substrates, for instance. Depending on the intended use and the outcome of the biological risk analysis, other biological effects, such as those related to immunotoxicological responses might also be relevant [157]. In this context, one prominent immunotoxicological effect is inflammation, and tests addressing the degree and duration of acute inflammation as well as tests pertaining to chronic inflammation (e.g. in case of a foreign body reaction) might be useful in the design and development phase. At present, however, the technical specification ISO/TS 10993‐20 [157] merely describes ideas of tests so that a future goal is to develop adequate test protocols in this space. Advanced analysis of cell‐surface interactions can be enhanced using Scanning Electron Microscopy (SEM), which provides high‐resolution insight into cellular adhesion, orientation, migration, and morphology. SEM enables direct visualization of features such as cell spreading, flattening, membrane protrusions (e.g., filopodia and lamellipodia), and overall surface architecture, revealing how coated versus uncoated substrates influence cytoskeletal organization and cell‐surface interactions at the micro‐ and nanoscale [158, 159, 160].

In long‑term or high‑wear applications, additional exposure pathways must be considered. Mechanical abrasion, environmental degradation products as discussed previously, or “end‑of‑life” processes such as incineration can generate airborne particles or degradation products [136, 161, 162, 163]. These may reach the lungs through inhalation or the digestive system through accidental ingestion [137, 163]. For this reason, a comprehensive risk assessment must incorporate all potential exposure routes.

### 4.3 Environmental Considerations

Release of materials from coatings represents a central aspect when evaluating the environmental aspects of the coatings. Understanding release is therefore a key step during the assessment. A general overview of the possible exposure pathways to the environment can be seen in Figure 8. These pathways include direct release to air, abrasion by touching or environmental weathering, release into water during indoor cleaning or rainfall, and release to soil through the application of sludge which is caused by release into wastewater, removal during wastewater treatment and subsequent soil application of sludge.

**FIGURE 8:**
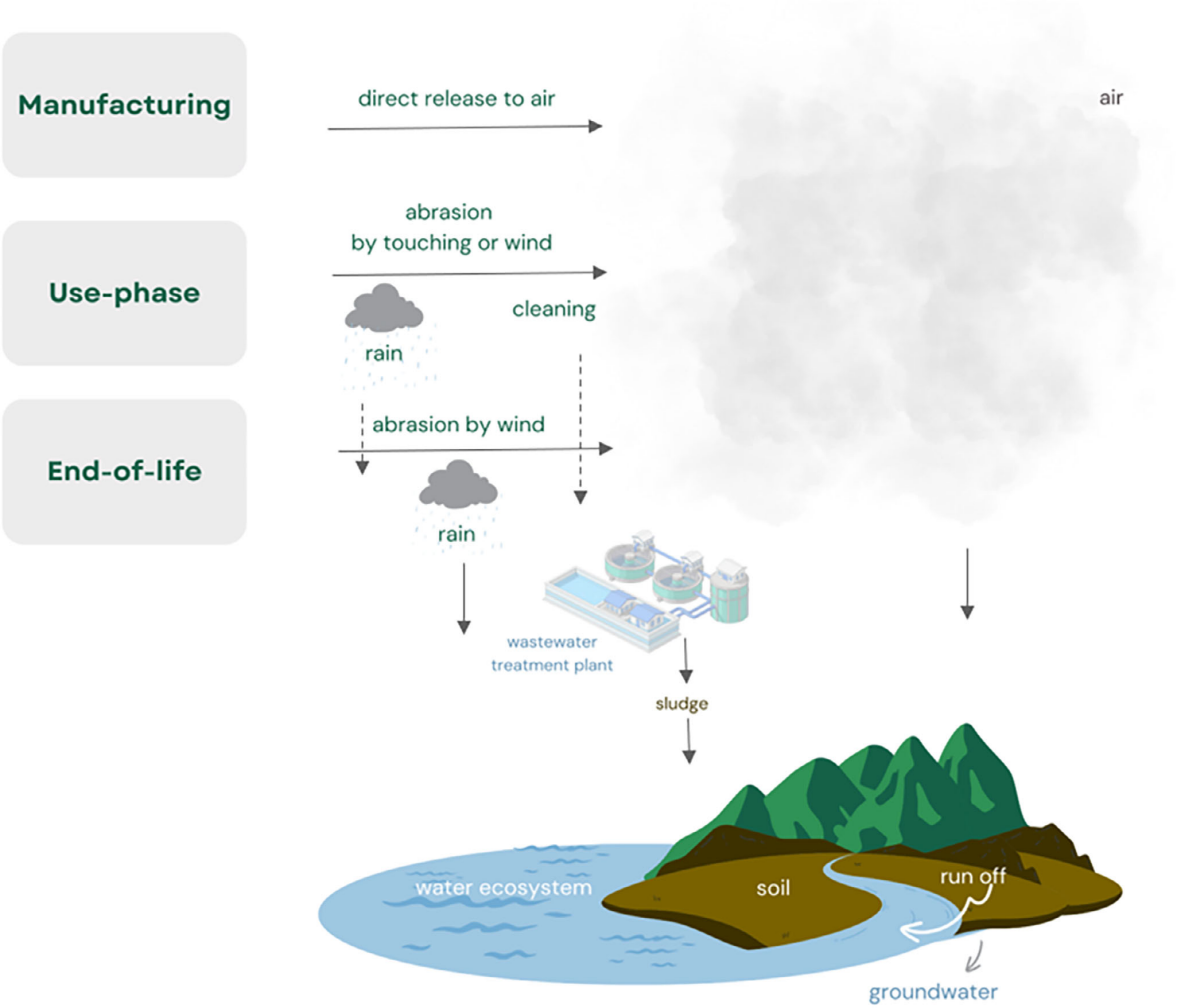
Exposure pathways of antimicrobial coatings to environmental compartments over their whole life cycle.

During manufacturing, direct release into air can occur, which is highly dependent on the characteristics of the active ingredient and the production technology. During the use phase, abrasion caused by environmental factors such as wind or rain may result in release to air and also leading to indirect exposure to other ecosystems. For instance, Keller et al. [164] stated that engineered nanomaterials, which are utilized in coatings, may be released into the environment as a result of weathering. Nowack et al. [165]. also highlighted that release of engineered nanomaterials from coatings is primarily caused by the product application, weathering and photodegradation. Another possible release pathway during the use phase is indoor release under increased airflow, for example, due to the wind produced by the movement of trains in an underground subway. One of the most relevant release pathways that should be emphasized, however, is cleaning during the use phase. As the coated surface is cleaned, particles may be transferred to the cleaning cloth [166]. In the case of the cloth being washed afterwards, the released particles can be transferred to wastewater treatment plants where a fraction of released materials may not be removed by a conventional wastewater treatment plant [167]. Depending on country‐specific waste management practices, sludges containing the removed active ingredients may ultimately be applied to or disposed of on soil. A modeling study for coatings, paints and pigments has shown that during the use phase, 35% of the released material enters soil, 60% is discharged to wastewater treatment plants, and 1% is released to the atmosphere [164].

End‐of‐life may represent the main release stage if coatings are not properly managed. For instance, if a train is left outdoors at a scrapyard, the internal coatings may gradually release active ingredients into the environment over time. Through rain, wind, and other environmental factors, these active ingredients may end up in soil, sediments, air, and water. For engineered nanoparticles, Sun et al. [168] stated that sediments have the highest concentration of engineered nanoparticles, followed by sewage treatment plant‐sludge treated soil, untreated soil and surface water, and finally air. Keller et al. [164] indicated that the majority of nanomaterials end up in landfills, with soils representing the second main sink. Leachate from landfills may also serve as a pathway for the release to soil and groundwater [167].

Once released, these substances may persist, bioaccumulate, or exert toxic effects on non‐target organisms, including aquatic organisms, plants, and invertebrates [169, 170]. To evaluate these effects, a combination of laboratory‐based ecotoxicological assays and environmental fate studies is typically employed. Acute and chronic toxicity can be assessed using standardized test organisms representing both aquatic and terrestrial environments. Aquatic organisms typically include water flea (*Daphnia magna*) for invertebrate toxicity [171, 172], duckweed (*Lemna minor*) for primary producer growth inhibition in aquatic macrophytes [173, 174], fish embryos (e.g., *Danio rerio*, zebrafish) for early life‐stage effects [175], and the ciliate protozoan *Tetrahymena thermophila*, which is widely used as a sensitive unicellular model for rapid screening of cytotoxic and metabolic impacts [176]. Terrestrial toxicity can be evaluated using the isopod *Porcellio scaber*, which serves as a model detritivore in soil ecotoxicology for assessment of toxic and sub‐toxic, stress endpoints [177].

Moreover, continuous low‐level exposure to biocides in the environment may contribute to the development of antimicrobial resistance, further underscoring the importance of designing coatings that balance efficacy with environmental responsibility [178, 179].

## 5 Safe and Sustainable by Design Approaches

The European Green Deal, launched in 2019, includes a variety of policy initiatives to achieve climate neutrality by 2050 through a green transition [180]. One of its key commitments is the zero pollution ambition for a toxic‐free environment, as part of which the Chemicals Strategy for Sustainability [181] was published. One of the actions of the strategy focuses on innovation to support the production and use of chemicals that are inherently safe and sustainable by design [181]. To facilitate the innovation process for chemicals and materials, the Safe and Sustainable by Design (SSbD) framework was published in 2022 [24] and updated in 2025 [182]. The main aims of the framework include i) steering innovation to support the green industrial transition; ii) substituting or minimizing the production and use of substances of concern, in line with and beyond regulatory requirements; and iii) minimizing or eliminating the impacts on health, climate, and the environment across the life cycle of chemicals, materials, and products, iv) guiding the criteria development for safe and sustainable chemicals/materials, and v) enabling their comparative assessment for specific functions or applications in the context of safety and sustainability [24]. The first version of the SSbD‐framework was followed by a methodological guidance [183], a report about challenges and proposed possible actions [184], and more recently a revised framework [182].

The current version of the SSbD framework consists of the following components: intended innovation—design/redesign, scoping analysis, SSbD scenario building, safety and sustainability assessment, SSbD evaluation, and documentation. Sector‐specific needs may be addressed through adaptations or additions to the current SSbD Framework. Fantke et al. [185]. stated that most case studies from funded SSbD projects focus on textiles, electronics, automotive, and construction materials., and further emphasized that broad coverage of economic sectors is important for identifying and addressing sector‐specific requirements and challenges. Wohlleben et al. [186]. also pointed out that the SSbD‐assessment can be made more efficient by developing sector‐specific tools or guidance. Some examples from the literature with focus on a specific sector or application areas include the pharmaceutical sector by Puhlmann et al. [187], and Sorani et al. [188], nanotechnology by Furxhi et al. [189], and advanced materials, with a particular focus on the safety assessment component of the SSbD by Pomar Portillo et al. [144]. Recently, a biocide‐specific SSbD Framework was also published to better assess materials with biocidal properties, which are also used in antimicrobial coatings [135]. The general overview of the biocide‐specific framework can be seen in Figure 9. Briefly, the inclusion of specific questions and central aspects tailored for the chemical/material is suggested along with the consideration of severity and possibility of accidents, assessment of societal needs and impacts to account for the benefits provided by the intended functionality; which relies on intended toxic effects toward target organisms and the establishment of a feedback loop to support decision‐making mechanisms.

**FIGURE 9:**
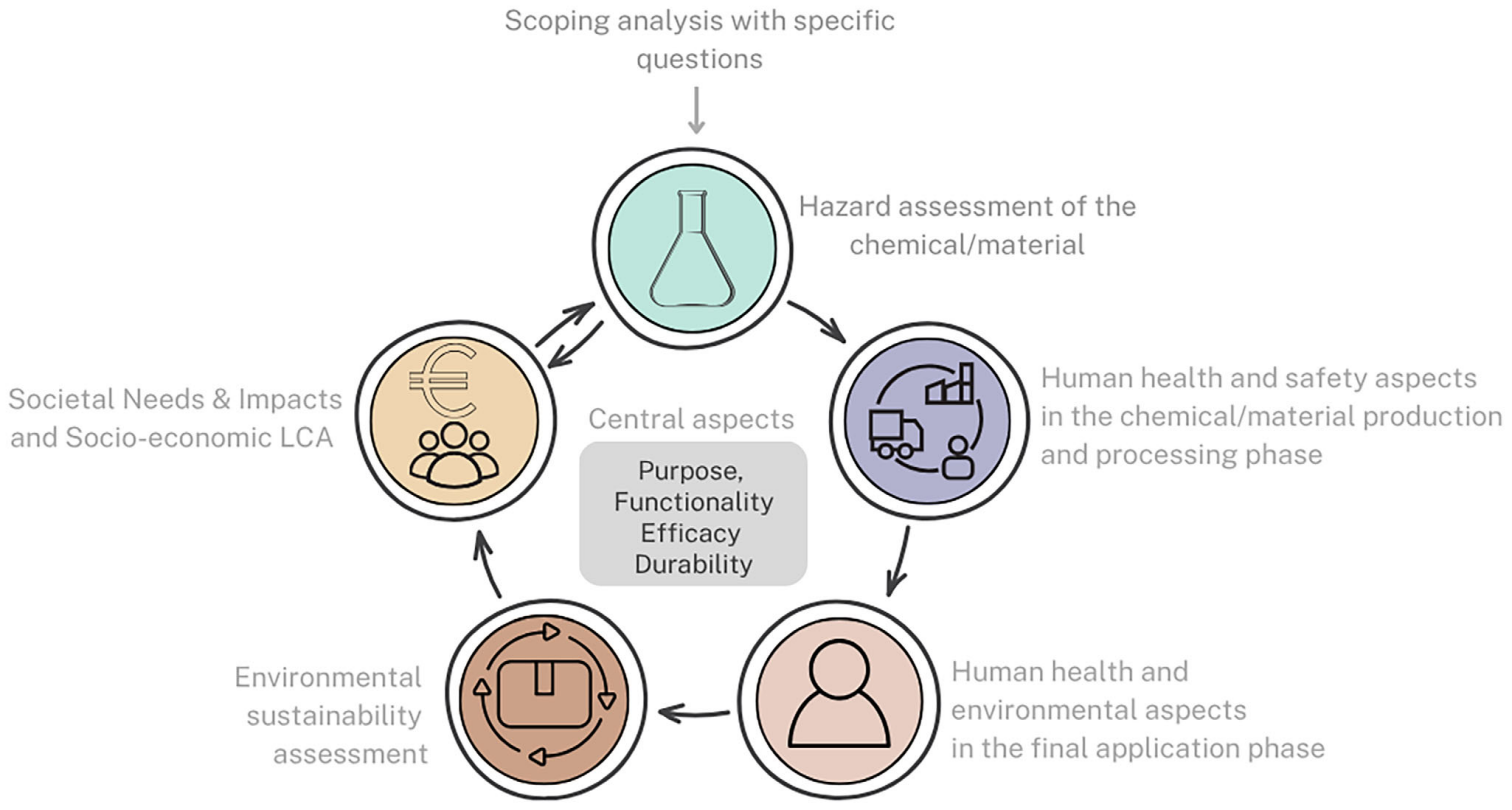
Biocide Specific SSbD (Adapted from [135], Copyright 2025, Elsevier, under the terms of the Creative Commons CC‐BY).

To begin with safety considerations, one of the most relevant hazard classifications for initial assessment is skin sensitization or skin corrosion for antimicrobial coatings on frequently touched surfaces. As the technology readiness level (TRL) level develops, additional hazard classifications may be considered, regardless of their regulatory requirements. Since the use phase is highly relevant for frequently touched surfaces, it is crucial to consider chemical release, as abrasion may facilitate the release of active ingredients. This may play a significant role in risk analysis for humans and the environment during the use phase, as well as in life cycle assessment for sustainability. Juhl et al. [190] provided general recommendations on sustainability issues in the coating industry, including minimizing the release of toxic substances. Punz et al. [191]. pointed out that ion‐releasing materials can be effective for short‐term applications, however, due to their dissolution properties, they may not be suitable for long‐term uses such as workplace surface coatings, where frequent replacement would be necessary. Maintaining long cleaning intervals without compromising performance is therefore important. The durability of the material should be considered, as it affects the frequency of application, during which additional release and environmental impacts may occur. Juhl et al. [190] further pointed out that durability is a key factor in assessing the sustainability of coatings, as it enables the desired antimicrobial function to be maintained for as long as possible. One of the suggestions they provided for eco‐strategies in coatings is, therefore, to enhance performance and durability.

On the other hand, if the active ingredient is highly durable, it is also important to consider the impacts of the materials at the end‐of‐life. For instance, the feasibility of separating the active ingredient from the coating to support recycling or reuse options should be considered. Using biodegradable ingredients may provide a solution to these challenges; however, in that case, it is also important to consider the required cleaning or application intervals, as this may also have drawbacks (e.g. increased operational work could lead to a higher environmental impact). The minimum sufficient concentration is another factor to be considered. In some cases, similar efficacy may be achieved at lower concentrations of active ingredients, thereby reducing the amount of chemicals used. Even though this practice is not commonly implemented, incorporating such considerations into an SSbD assessment could enable or facilitate the use of lower quantities of chemicals [135].

When it comes to the sustainability assessment, Juhl et al. [190]. conducted an LCA of raw materials for protective coatings. Among the materials assessed, titanium dioxide and metallic zinc dust may be relevant for antimicrobial coatings for frequently touched surfaces. The main environmental impacts from titanium dioxide include mineral resource scarcity, fine particulate matter formation, terrestrial acidification, and global warming, and those from metallic zinc dust include human non‐carcinogenic toxicity, freshwater ecotoxicity and eutrophication, and mineral resource scarcity. The authors also highlighted the importance of considering the use phase by giving an example of the replacement of a solvent‐based coating by a water‐based coating without any compromise for durability and performance but a better performance in the LCA. Moreover, the selection of the functional unit is crucial, as it may strongly affect the usefulness of the results. Chaztipanagiotou et al. [192] assessed the SSbD of antimicrobial coatings with metal nanoparticles, and the authors pointed out the importance of defining the functional unit in LCA. A benefit‐based functional unit (e.g. keeping 10 cm^2^ of surface antimicrobial for 1 month [135]) was suggested to facilitate comparison with the benchmark and to derive more useful insights.

Although the use phase is important when assessing the environmental impacts of a coating, it is worth considering the production methods as well. Chaztipanagiotou et al. [192] highlighted that metal nanoparticles may be released during manufacturing from the collection filter. Green synthesis methods may provide solutions not only for avoiding potential negative effects associated with the release but also for addressing other challenges related to the synthesis of nanoscale advanced materials such as instability under severe conditions, potential chronic toxicity due to bioaccumulation, and challenges with recycling [191]. On the other hand, innovative production methods may result in lower environmental impacts. Although specific examples for frequently touched surfaces could not be found in the literature, such examples are available for other types of coatings. For instance, Malvi et al. [193]. suggested a “nano‐island” technology for stainless steel coatings. While the traditional methods may release excessive silver ions, and may also affect non‐target organisms, their suggested technology minimizes Ag usage, therefore contributing to SSbD. Bhat et al. [194] also suggested bio‐based plasma‐assisted coatings for textiles, since conventional coating methods may have significant environmental and health risks. In addition, some conventional methods, such as padding and exhaustion, often involve high water usage and considerable energy for drying and curing. The authors stated that bio‐based plasma‐assisted coatings for textiles may promote sustainable applications by integrating dry plasma processes, minimizing chemical usage, and ensuring work and user safety.

We suggest considering tailored strategies based on the specific use case of the active ingredient in combination with the material used for frequently touched surfaces, taking into account the approaches mentioned above, as well as any additional methods that may arise from the type and properties of the coatings.

## 6 Conclusions and Future Perspectives

Antimicrobial coatings for high touch surfaces have attracted growing interest as a complimentary strategy to reduce microbial contamination across a broad range of surfaces. Significant progress has been made in antiadhesive, contact killing, release‐based, light‐activated and hybrid coating strategies, and many of these shows promising efficacy under controlled laboratory conditions. However, strong initial efficacy alone is not sufficient to establish practical value. Despite the large numbers of reported concepts, relatively few studies have shown whether these materials remain effective, durable and applicable under the conditions in which they are intended to be used. The key challenge for the field is therefore not only developing new antimicrobial concepts but generating coatings that are genuinely fit for the purpose in real world high touch environments.

From a material design perspective, the authors scored the efficacy and safety of each coating strategy using a common rubric to identify where future efforts should focus (Figure 10, details in Tables S1–S3). This expert elicitation suggests that efficacy is not the bottleneck for next generation coatings. Instead, safety and real‐world performance in a defined use case will distinguish them. Because these coatings are designed for broad spectrum lethality, a certain cytotoxicity and ecotoxicity is inherent to their function. Release is thus the key element, because whether and how the active agent leaves the coating governs human toxicity, environmental burden and resistance risk.

**FIGURE 10:**
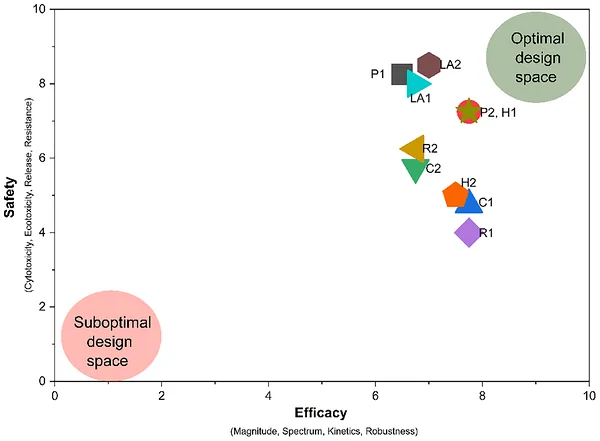
Expert elicitation of the efficacy and safety of the antimicrobial coating strategies for high touch surfaces. Each strategy was scored using four efficacy criteria (magnitude, spectrum, kinetics, robustness) and four safety criteria (cytotoxicity, ecotoxicity, release and resistance). Values are unweighted means of respective criteria, averaged across authors. Marked areas indicate optimal and suboptimal design spaces as a visual guide. P1: low‐adhesion superhydrophobic and micro/nanostructured surfaces; P2: slippery liquid‐infused porous surfaces (SLIPS); C1: immobilized metal contact‐active coatings; C2: immobilized cationic biocide coatings; R1: continuous ion release; R2: stimuli‐responsive/controlled release; LA1: photocatalytic coatings; LA2: photosensitizer‐based coatings; H1: hybrid combinations without a released biocide; H2: hybrid combinations including a releasing component. Details of the scoring rubric are provided in the Tables S1–S3.

Release‐based systems, especially those with uncontrolled kinetics, require careful evaluation. In our view, thin, non‐depleting and re‐activatable coatings, such as light‐activated systems, are a safer alternative. However, their activity depends on light availability in the intended use case, and their safety depends on preventing the loss of active agent through wear and cleaning. Because the active agent remains in the coating during use, its fate at end‐of‐life must also be considered. Passive antiadhesive strategies, on the other hand, do not kill microorganisms but can add value as complementary components of hybrid coatings.

A second priority is efficacy testing that better reflects real‐life conditions. Testing should move from idealized laboratory conditions toward application‐relevant protocols, including worst‐case scenarios. It should also become more comparable across coating types, for example through standardized illumination conditions for light‐activated systems and standardized approaches to quantify release from thin films. In addition, although these coatings are intended to act broadly, breadth of activity is inconsistently demonstrated across the strategies discussed. Efficacy is reported far more often against bacteria than against fungi or viruses, although all three contribute to transmission via high‐touch surfaces. Claims of broad‐spectrum activity should therefore be interpreted as reflecting the specific organisms tested rather than general antimicrobial breadth, and future studies should include the organisms most relevant to the intended use case. Ultimately, efficacy data should support realistic claims about performance in use, rather than only proof‐of‐concept activity.

Safety toward both humans and the environment also needs to become more central. Standardized in vitro cytotoxicity assays are available and accepted by regulatory bodies, but methods that capture realistic human exposure routes, such as dermal contact and inhalation, remain scarce. Future research should prioritize standardized, exposure‐relevant protocols for quantitative direct‐contact assessment and for inflammation‐related responses, where current guidance remains incomplete. Because many coatings are designed for long‐term use, chronic and low‐dose toxicity endpoints also deserve more attention.

The same release processes that drive user exposure also determine how antimicrobial components enter the environment during use (e.g., through cleaning) and at end of life. This is particularly relevant for particle‐containing and release‐based coatings, where leaching, degradation, abrasion or disposal may occur. Knowledge of the accumulation, persistence, uptake and ecological impacts of these released materials remains limited. Future work should therefore adopt a life cycle perspective that links antimicrobial performance with release behavior, human and environmental exposure pathways, and possible impacts on organisms.

Beyond technical performance, regulatory preparedness and market entry are increasingly decisive. Approval pathways, evidence requirements and compliance expectations may differ substantially with the mode of action. This creates uncertainty for developers and slows the translation of novel technologies that may be less toxic and more environmentally friendly. Progress will therefore also depend on clearer and more proportionate assessment pathways aligned with mode of action, intended use and long‐term risk–benefit considerations. Stronger communication between researchers, manufacturers, regulatory bodies and end users will also be needed.

Taken all together, these challenges highlight the importance of integrating the SSbD approach from the early stages of developing antimicrobial coatings for high touch surfaces. This may provide guidance specifically for the selection of active ingredients and materials, the determination of the mode of action, manufacturing methods and planning for end‐of‐life phases while considering release aspects. In practice, this highlights the importance of balancing antimicrobial function with durability, non‐toxicity, as well as environmental compatibility to reach the maximum efficiency for the intended function. The successful deployment of SSbD depends strongly on collaboration across disciplines and sectors to ensure that coatings not only perform under laboratory conditions but are also used in a safe and sustainable way in their intended settings.

## Author Contributions

**Bernd Nowack**: conceptualization, supervision, funding acquisition, project administration, Writing – review and editing. **Nurshen Mutlu**: conceptualization, methodology, investigation, visualization, writing – original draft. **Peter Askew**: writing – review and editing. **Damjana Drobne**: writing – review and editing. **Stefan Fischer**: writing – review and editing. **Gillian Iredale**: writing – review and editing. **Pascal Kock**: writing – review and editing. **Veno Kononenko**: writing – review and editing. **Susanne Kurz**: writing – review and editing. **Patrick Lichtner**: writing – review and editing. **Abby Marchant**: writing – review and editing. **Philipp Meier**: writing – review and editing. **James Redfern**: writing – review and editing. **Giacomo Reina**: writing – review and editing. **Simone Schulte**: writing – review and editing. **Juliane Spohn**: writing – review and editing. **Merve Tunali**: writing – review and editing. **Peter Wick**: writing – review and editing. **Nihal Engin Vrana**: writing – review and editing, project administration, funding acquisition.

## Conflicts of Interest

The authors declare no conflicts of interest.

## Supporting information




**Supporting File**: advs78521‐sup‐0001‐SuppMat.docx

## Data Availability

Data sharing not applicable to this article as no datasets were generated or analyzed during the current study.
