# Supplementary material for: Next Generation Antimicrobial Coatings for High Touch Surfaces: Application, Benefits, Risks, Environmental Impacts and Sustainability Aspects
Source: Adv Sci (Weinh). 2026 Nov 10:e78521. Online ahead of print. doi: 10.1002/advs.78521 (PMC13752236; doi:10.1002/advs.78521)
Supplement: Supplementary file 1 [file ADVS-9999-e78521-s001.docx]

Supporting Information

**Next Generation Antimicrobial Coatings for High Touch Surfaces: Application, Benefits, Risks, Environmental Impacts and Sustainability Aspects**

*Nurshen Mutlu^1^, Peter Askew^2^, Alexander J Cunliffe^3^, Damjana Drobne^4^, Stefan Fischer^5^, Gillian Iredale^2^, Pascal Kock^5^, Veno Kononenko^4^, Susanne Kurz^6^, Patrick Lichtner^6^, Abby Marchant^2^, Philipp Meier^7^, James Redfern^3^, Giacomo Reina^7^, Simone Schulte^5^, Juliane Spohn^6^, Merve Tunali^1^, Peter Wick^7^, Nihal Engin Vrana^8^, Bernd Nowack^1*^*

^1^ Empa - Swiss Federal Laboratories for Materials Science and Technology, Technology and Society Laboratory, 9014 St. Gallen, Switzerland

2 Industrial Microbiological Services Ltd. (IMSL), Pale Lane, Hartley Whintey, Hants RG27 8DH, United Kingdom

^3^ School of Biological and Chemical Sciences, Manchester Metropolitan University, Manchester, United Kingdom

^4^ Department of Biology, Biotechnical Faculty, University of Ljubljana, 1000 Ljubljana, Slovenia

^5^ Microbial Effects, Coating Additives, Evonik Operations GmbH, Essen 45127, Germany

^6^ Biological Materials Analysis Group, Fraunhofer Institute for Ceramic Technologies and Systems (IKTS), 04103 Leipzig, Germany

^7^ Empa - Swiss Federal Laboratories for Materials Science and Technology, Laboratory for Nanomaterials in Health, 9014 St. Gallen, Switzerland

^8^ SPARTHA Medical, CRBS, 67000, Strasbourg, France

Correspondence: Bernd Nowack ([nowack@empa.ch](mailto:nowack@empa.ch))

This Supporting Information explains how the efficacy-safety comparison in Figure 10 was made. Tables S1 and S2 show the scoring criteria and scales. Table S3 lists the coating classes and the reference systems used as examples of each class. The scores were obtained by expert elicitation: the individual authors scored every class separately using the rubrics, and the final score is the average of these scores. The authors were asked to score the class as a whole, based on what is usually reported for it, and not the best system in it. The scores are only meant for comparing the classes with each other.

Table S1. Efficacy scoring rubric. The authors were asked to score each subcriterion (E1-E4) from 0 to 2.5, following the descriptions in the table. The four sub-scores were added to give the efficacy score (0-10).

| **Code** | **Subcriteria** | **What it captures** | **0** | **0.5-1.0** | **1.5** | **2.0** | **2.5** |
| --- | --- | --- | --- | --- | --- | --- | --- |
| **E1** | **Magnitude of activity** | Extent of microbial reduction typically achieved by this class. For passive classes, adhesion or biofilm coverage is used instead of log reduction." | No reduction | < 1 log  (or <50% adhesion reduction) | 1 to <2 log  (or 50 to <90% adhesion reduction) | 2 to <4 log  (or 90 to <99% adhesion reduction) | ≥4 log  (or ≥99% adhesion reduction) |
| **E2** | **Spectrum breadth** | Number of microbial targets with demonstrated activity: Gram-positive, Gram-negative, fungi/yeasts, viruses, and established biofilm. | None demonstrated | 1 target | 2 targets | 3 targets | 4-5 targets |
| **E3** | **Kill/inactivation kinetics** | Contact time needed to reach the reported reduction. Critical for high-touch surfaces, which are re-contaminated fast. For passive classes the criterion is how long the reduction in adhesion lasts. | Not reported | >24 h (passive: <1 h) | >6 to ≤24 h (passive: 1 to <6 h) | >1 to ≤6 h (passive: 6 to <24 h) | ≤1 h (passive: ≥24 h) |
| **E4** | **Real-world robustness** | Retention of activity under realistic conditions: ambient or indoor light rather than intense UV, organic soiling, humidity, repeated cleaning, abrasion, and service life. | Ideal lab conditions only | One realistic stressor, large activity loss | One stressor, moderate loss | Two or more stressors with activity retained | Validated in situ or field-tested with activity retained |

Note: For passive classes, E1 and E3 were scored on the reduction in adhesion and on how long the effect lasts, instead of log reduction and kill time.

Table S2. Safety scoring rubric. Authors were asked to score each sub-criterion (S1-S4) from 0 to 2.5, following the descriptions in the table. The four sub-scores were added to give the safety score (0-10).

| **Code** | **Subcriteria** | **What it captures** | **0** | **0.5-1.0** | **1.5** | **2.0** | **2.5** |
| --- | --- | --- | --- | --- | --- | --- | --- |
| **S1** | **Human toxicity** | Toxicity to human cells (keratinocytes, fibroblasts, lung epithelium) from the coating or its released part | Marked cytotoxicity | Significant cytotoxicity | Moderate or borderline | Low cytotoxicity | No cytotoxicity |
| **S2** | **Ecotoxicity and persistence** | Aquatic and terrestrial toxicity, environmental persistence and bioaccumulation of released species, including wash-off during routine cleaning and release at end of life. This reflects the harm caused by the released species, not the coating's loss of performance. | Persistent, bioaccumulative, high toxicity | High toxicity or persistent | Moderate concerns | Low concerns / rapidly degrade species | No persistent mobile species released |
| **S3** | **Release behavior** | How fast the active substance leaves the coating, and how controllable that is. The main difference is between a system that releases continuously and an active substance that either stays bound to the surface or is generated on the spot and does not persist. This covers intentional release-based systems | Continuous, uncontrolled leaching | Continuous but slowed or encapsulated | Controlled or stimulus-triggered release | Minimal release; active species mostly immobilized | No release: active immobilized or transient |
| **S4** | **Resistance and secondary risks** | Whether the coating could lead resistant microorganisms, including resistance to other agents at the same time. | Known resistance selection | Documented resistance concern | Plausible but unquantified concern | Low risk; multi-target mechanism | Multi-target, insignificant secondary hazard |

Table S3. Coating classes, antimicrobial strategies and reference systems. The reference systems are examples of each class.

| **Code** | **Class** | **Strategy** | **Reference system** |
| --- | --- | --- | --- |
| **P1** | Low adhesion / superhydrophobic /micro-nanostructured surface | Passive (Antiadhesive) | Superhydrophobic PEG and PVA coatings; hierarchical wrinkled polysiloxane nanostructures |
| **P2** | Liquid-infused slippery surfaces (SLIPS) | Passive (Antiadhesive) | Slippery liquid-infused porous surfaces |
| **C1** | Immobilized metal contact-active coatings | Contact-killing | Surface-immobilized silver and copper. If release of metal ions is the main reported mechanism, the system is placed in R1 instead. |
| **C2** | Immobilized cationic biocide coatings | Contact killing | Immobilized quaternary ammonium compounds; sprayable quaternary small molecules. |
| **R1** | Continuous ion release | Released-based | Ag^+^ release from immobilized mesoporous silica nanoparticles; Ag-doped phosphate glass additives in powder coating on aluminum. Systems where release into the surroundings is the main antimicrobial mechanism are placed here. |
| **R2** | Stimuli responsive /Controlled release | Released-based | Smart-release Cu^2+^ -loaded cation-exchange resin dispersed in polymer binder |
| **LA1** | Photocatalytic coating | Light activated | TiO_2_ and doped TiO_2_ formulations active under daylight |
| **LA2** | Photosensitizer-based coatings | Light activated | Organic photosensitizers; upconverter-containing systems |
| **H1** | Hybrid combinations without a released a releasing component | Hybrid | Quaternized chitosan (contact-killing) + acetal-based pH-responsive self-cleaning crosslinker + TiO_2_ with reduced graphene oxide for visible-light activation |
| **H2** | Hybrid combinations with releasing component | Hybrid | Nano silica resin (Li/K silicates) + silver nitrate + N-alkylpropylaminoglycine; Ag and TiO_2_ nanoparticle systems |
